# WRKY23 is a component of the transcriptional network mediating auxin feedback on PIN polarity

**DOI:** 10.1371/journal.pgen.1007177

**Published:** 2018-01-29

**Authors:** Tomáš Prát, Jakub Hajný, Wim Grunewald, Mina Vasileva, Gergely Molnár, Ricardo Tejos, Markus Schmid, Michael Sauer, Jiří Friml

**Affiliations:** 1 Institute of Science and Technology (IST), Klosterneuburg, Austria; 2 Laboratory of Growth Regulators, Palacký University, Olomouc, Czech Republic; 3 Department of Plant Biotechnology and Bioinformatics, Ghent University and Center for Plant Systems Biology, VIB, Ghent, Belgium; 4 Facultad de Recursos Naturales Renovables, Universidad Arturo Prat, Iquique, Chile; 5 Department of Molecular Biology, Max Planck Institute for Developmental Biology, Tübingen, Germany; 6 Department of Plant Physiology, Umeå Plant Science Centre, Umeå University, Umeå, Sweden; 7 Department of Plant Physiology, University of Potsdam, Potsdam, Germany; Washington University in St. Louis, UNITED STATES

## Abstract

Auxin is unique among plant hormones due to its directional transport that is mediated by the polarly distributed PIN auxin transporters at the plasma membrane. The canalization hypothesis proposes that the auxin feedback on its polar flow is a crucial, plant-specific mechanism mediating multiple self-organizing developmental processes. Here, we used the auxin effect on the PIN polar localization in *Arabidopsis thaliana* roots as a proxy for the auxin feedback on the PIN polarity during canalization. We performed microarray experiments to find regulators of this process that act downstream of auxin. We identified genes that were transcriptionally regulated by auxin in an AXR3/IAA17- and ARF7/ARF19-dependent manner. Besides the known components of the PIN polarity, such as PID and PIP5K kinases, a number of potential new regulators were detected, among which the WRKY23 transcription factor, which was characterized in more detail. Gain- and loss-of-function mutants confirmed a role for WRKY23 in mediating the auxin effect on the PIN polarity. Accordingly, processes requiring auxin-mediated PIN polarity rearrangements, such as vascular tissue development during leaf venation, showed a higher *WRKY23* expression and required the WRKY23 activity. Our results provide initial insights into the auxin transcriptional network acting upstream of PIN polarization and, potentially, canalization-mediated plant development.

## Author summary

The plant hormone auxin belongs to the major plant-specific developmental regulators. It mediates or modifies almost all aspects of plant life. One of the fascinating features of the auxin action is its directional movement between cells, whose direction can be regulated by auxin signaling itself. This plant-specific feedback regulation has been proposed decades ago and allows for the self-organizing formation of distinct auxin channels shown to be crucial for processes, such as the regular pattern formation of leaf venation, organ formation, and regeneration of plant tissues. Despite the prominent importance of this so called auxin canalization process, the insight into the underlying molecular mechanism is very limited. Here, we identified a number of genes that are transcriptionally regulated and act downstream of the auxin signaling to mediate the auxin feedback on the polarized auxin transport. One of them is the *WRKY23* transcription factor that has previously been unsuspected to play a role in this process. Our work provides the first insights into the transcriptional regulation of the auxin canalization and opens multiple avenues to further study this crucial process.

## Introduction

The phytohormone auxin plays a key role in many aspects of a plant’s life cycle. A unique attribute of auxin is its polarized, intercellular movement that depends, among other components, on the polarly localized PIN-FORMED (PIN) auxin exporters [1–3]. The so-called canalization hypothesis proposes that auxin acts also as a cue in the establishment of new polarity axes during the polarization of tissues by the formation of self-organizing patterns due to the formation of narrow auxin transport channels driven by the polarized auxin carriers from an initially broad domain of auxin-transporting cells [4–6]. Canalization has been implied to mediate multiple key plant developmental processes, including formation of new vasculature [7], regeneration after wounding [8, 9], and competitive control of apical dominance [10–12]. Whereas the molecular details of canalization are largely unknown, the key constituents are *(i)* the feedback regulation of the auxin transport directionality by auxin and *(ii)* the gradual concentrating and narrowing of auxin channels [4]. The auxin feedback on the transport directionality can be realized by the auxin impact on the PIN polarity [8] and might be related to an auxin effect on clathrin-mediated internalization of PIN proteins [13, 14], but the connection is still unclear [15]. Presumably, this feedback regulation of the PIN repolarization also plays a role in the establishment of the embryonic apical-basal axis [16, 17], during organogenesis [18], and termination of shoot bending responses [19].

Auxin feedback on the PIN polarity can be experimentally approximated by PIN polarity rearrangements after auxin treatment of *Arabidopsis thaliana* roots. Under standard conditions, PIN1 is localized at the basal (root-ward) sides of endodermal and pericycle cells and cells of the vascular tissue [20], whereas PIN2 exhibits a basal polarity in the young cortex cells, but an apical (shoot-ward) polarity in epidermal cells [21, 22]. After treatment with auxin, PIN1 changes from predominantly basal to also inner-lateral in endodermal and pericycle cells, whereas PIN2 undergoes a localization shift from the basal to also outer-lateral side of cortex cells [8]. The exact molecular mechanism and biological significance of this effect is unclear, but it has so far successfully served as easy, experimentally tractable proxy for auxin feed-back on PIN polarity [8]. It depends on the transcriptional SCF^TIR1^-Aux/IAA-ARF auxin signalling pathway [23]. In brief, upon auxin binding to the TIR1/AFB receptor family, transcriptional repressors and co-receptors of the Aux/IAA class are degraded, in turn releasing auxin response transcription activators of the ARF family [24, 25].

In a heat-shock (HS)-inducible *HS*::*axr3-1* line expressing a mutated, nondegradable version of the IAA17 transcriptional repressor [25, 26], as well as in the *arf7 arf19* double mutant defective for these two functionally redundant transcriptional activators expressed in primary roots [27], auxin is no longer effective in mediating PIN polarity rearrangements in the root meristem [8]. These results suggest that transcriptional auxin signalling regulates the cellular abundance of so far unknown regulators, which, in turn, modify subcellular sorting or trafficking pathways and other polarity determinants, ultimately leading to changes in the polar PIN distribution.

In this work, we carried out an expression profiling experiment in *Arabidopsis* roots to identify potential regulators of the PIN polarity that are transcriptionally regulated by auxin signalling. We identified several novel regulators and characterized in more detail the transcription factor WRKY23 and its role in auxin-mediated PIN polarization, thus providing initial insights into a molecular mechanism of the auxin feedback on the directional auxin flow–one of the key prerequisites of canalization.

## Results

### Microarray-based identification of components mediating auxin impact on PIN polarity

**Fig 1:**
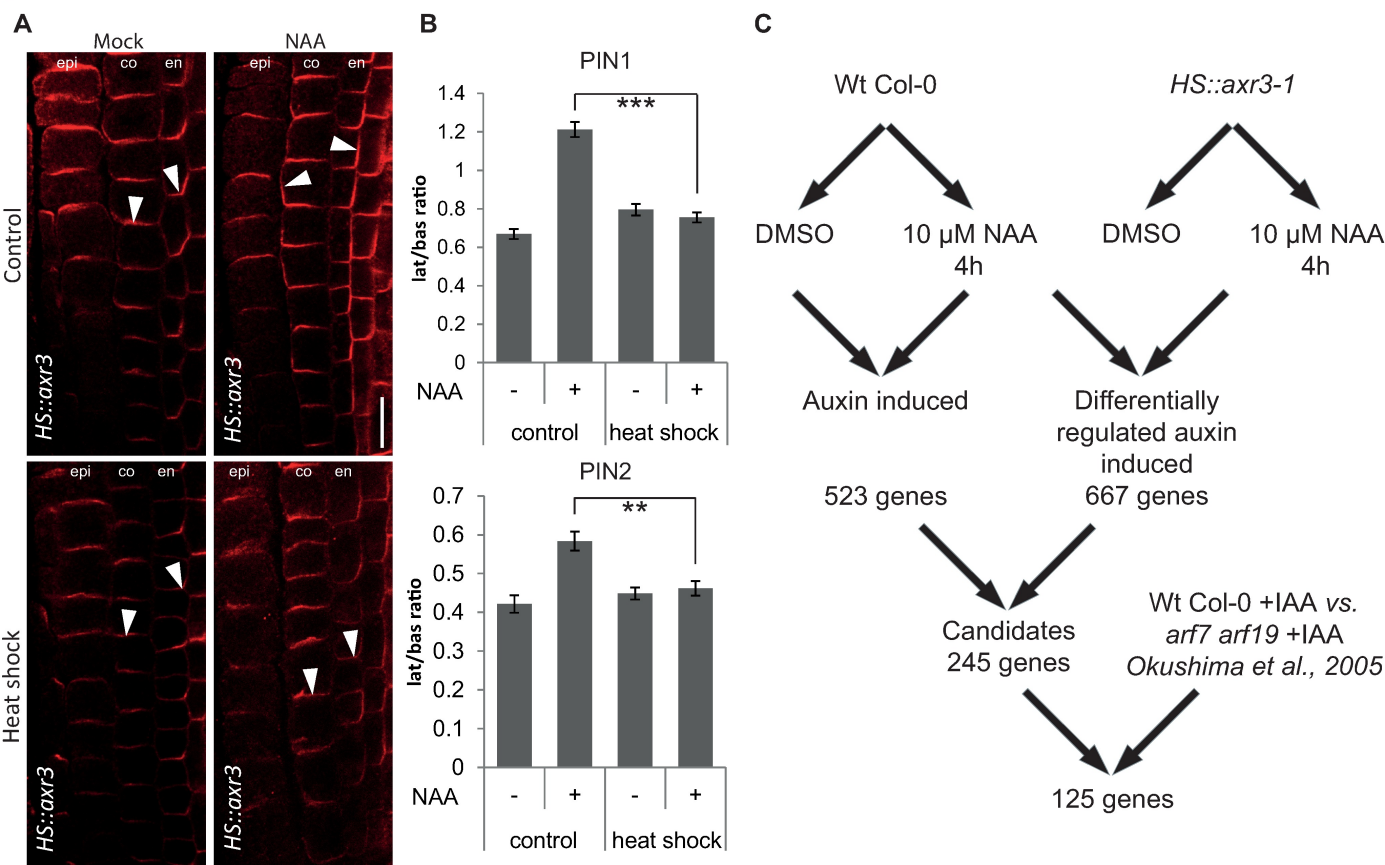
Putative transcriptional components of the auxin-mediated PIN polarization. **(**A) Simultaneous immunolocalization of PIN1 and PIN2 in *HS*::*axr3-1* plants. Heat shock-induced overexpression of *axr3-1* abolishes lateral PIN relocation after auxin (4 h, 10 μM NAA) treatment, confirming dependence on the SCF^TIR1^-Aux/IAA-ARF signalling pathway. Arrowheads highlight representative examples of PIN localization in the respective tissues and treatments (PIN1 in endodermis and PIN2 in cortex). Bar = 10 μm. epi, epidermis; co, cortex; en, endodermis. (B) Quantitative evaluation of (A), confirming reduced auxin-dependent relocation of PIN1 (top) and PIN2 (bottom) in the induced *HS*::*axr3-1* line. Graph shows mean ratio of lateral-to-basal signal intensity of PIN1 in endodermal and PIN2 in cortex cells. Error bars indicate standard error. A One-Way ANOVA test compared marked sets of data. (** *p*<0.01; *** *p*<0.0001; *n*>35 cells corresponding to a minimum of 10 roots per treatment and experiment were imaged under comparable conditions). Experiments were carried out at least 3 times; one representative experiment is presented. (C) Scheme of the microarray experiment and analysis strategy.

The rationale behind the microarray approach was to search for genes that were (*i*) regulated by auxin in roots under conditions when auxin changes PIN polarity and (*ii*) their auxin regulation is mediated by the IAA17 (AXR3) transcriptional repressor. First, to look for auxin-induced genes, we matched data from NAA-treated and untreated heat-shocked wild type (WT) Columbia-0 (Col-0) control seedlings and found 523 auxin-induced genes, with a minimum of two-fold difference. As in the *HS*::*axr3-1* line under the same conditions auxin fails to induce PIN polarity changes (Fig 1A and 1B) [8], we compared heat-shocked and auxin-treated Col-0 seedlings to similarly handled *HS*::*axr3-1* seedlings, expressing the auxin-resistant version of IAA17 (AXR3) and we identified 667 genes (Fig 1C). The overlap of this set with the 523 auxin-induced genes yielded 245 genes induced by auxin and regulated downstream of IAA17 (S1 Table), including *PATELLIN2* and *PATELLIN6* that encode phosphatidylinositol transfer proteins, concomitantly characterized to be crucial for the regulation of embryo and seedling patterning in *Arabidopsis* [28]. Further comparison with published microarray data on *arf7 arf19* mutant seedlings [29], which are also ineffective in rearranging the PIN polarity [8], yielded a final list of 125 genes (S2 Table), of which some had previously been found to be involved in PIN polarity regulation, including the AGC3 kinase PINOID (PID). and its homologs WAG1 and WAG2 are known to phosphorylate PIN proteins [30], contributing to the control of their polar distribution [31–33]. Nevertheless, overexpression of *PID* was shown to be dominant over the auxin-induced PIN lateralization [8]. Another identified candidate with a known role in the PIN polar distribution was the phosphatidylinositol-4-phosphate 5 kinase PIP5K1. This protein, together with its close homolog PIP5K2, is enriched on basal and apical membrane domains and they are required for PIN trafficking [34, 35] and localization [36, 37]. Other candidates for polarity determinants include several previously known players in auxin-mediated plant development, such as RUL1, a leucine-rich repeat receptor-like kinase regulating cambium formation, a process linked to PIN polarity control [38].

Auxin-dependent PIN lateralization in the root meristem requires a rather prolonged auxin treatment [8], hinting at the involvement of a whole cascade of transcriptional processes. Therefore, we looked for additional auxin-induced transcription factor (TF) genes, which, based on their analogous behaviour in similar experiments and on their known functions, would be potential candidates for having a role in auxin-mediated development. The list of candidates contains *e*.*g*. *MINI ZINC FINGER1 (MIF1*), affecting auxin responses during ectopic meristem formation [39], but also *WRKY23*. *WRKY* genes belong to a plant-specific family of 72 TFs in *Arabidopsis*, typically associated with plant defense processes and plant-pathogen interactions [40]. These genes were named by a shared sequence motif of 60 amino acids containing a conserved domain of seven invariant amino acids (WRKYGQK) [41]. The WRKYGQK motif provides a high binding preference and contacts a 6-bp DNA sequence element–the W-box (/TTGACT/C) contained in target gene promoters [40, 42]. Distinct WRKY TFs have distinct selective binding preferences to certain W-box variants [43]. The role of WRKY23 has been established in plant defence processes during plant-nematode interactions, but also in auxin transport regulation by flavonol biosynthesis that affects root and embryo development. In *Arabidopsis* embryos, the *WRKY23* expression attenuates both auxin-dependent and auxin-independent signalling pathways toward stem cell specification [44–46]. In addition, *WRKY23* is unique within its gene family, because none of the other *WRKY* genes in these experimental conditions was responsive to auxin and, thus, present in the gene selection (S2 Table). In this work, we focused on one of the transcription factors fulfilling our selection criteria, and investigated the role of WRKY23-dependent transcriptional regulation in auxin-dependent PIN repolarization.

### *WRKY23* expression is regulated by auxin signalling

**Fig 2:**
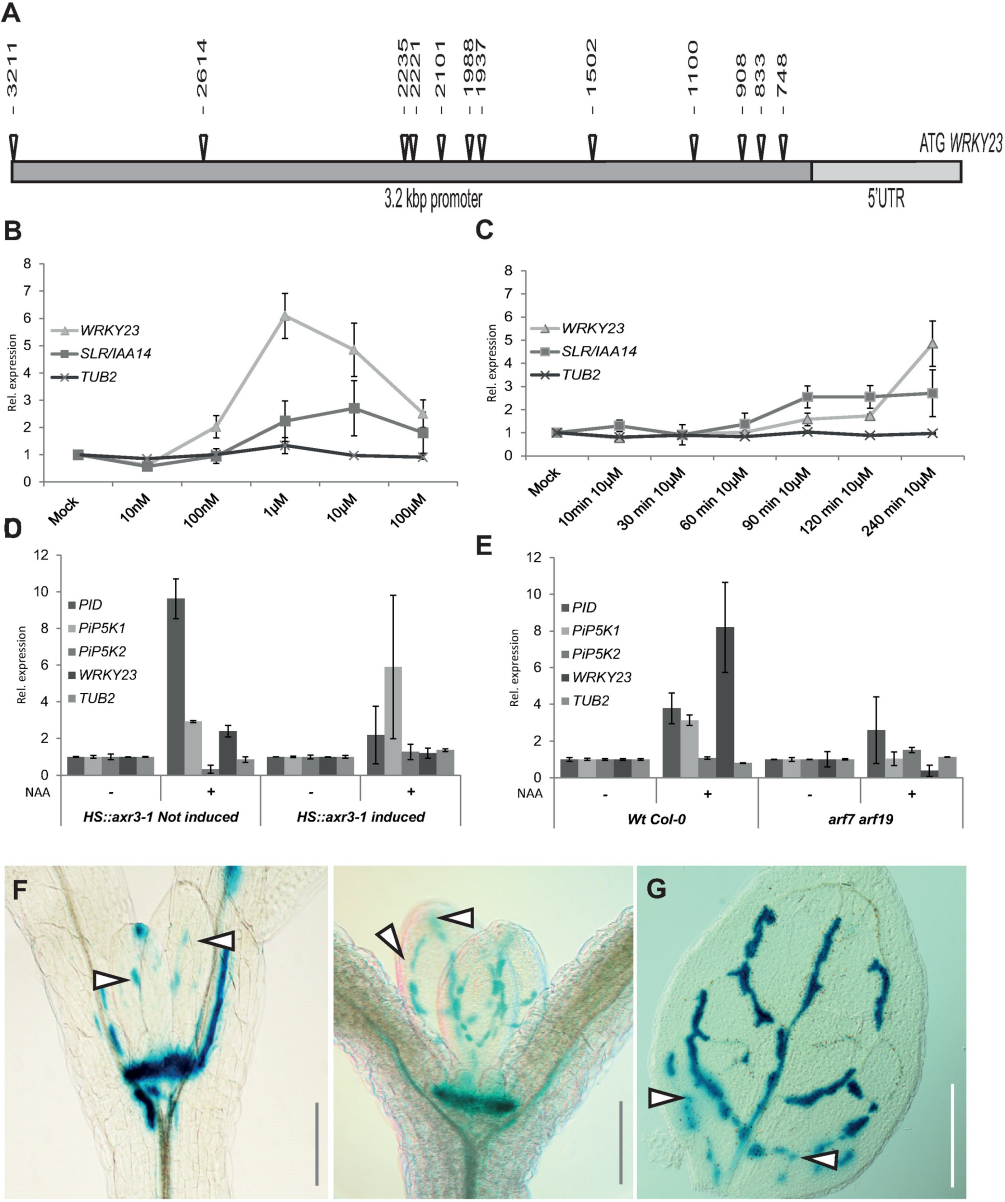
WRKY23 acts downstream of the Aux/IAA—ARF auxin pathway and marks developing vasculature. (A) Schematic depiction of WRKY23 promoter; AuxRE and AuxRE-like response elements are shown as triangles (B and C) *WRKY23* transcript levels depend on auxin dose and treatment time. qRT-PCR analysis of *WRKY23* expression after a 4 h treatment with different concentrations of NAA (B) and after different treatment times with 10 μM NAA (C). *TUB2* and *SLR/IAA14* are shown as negative and positive controls, respectively. Values represent relative fold change of expression. Error bars represent standard deviation (see Materials and Methods for detailed description). (D and E) *WRKY23* expression depends on the SCF^TIR1^-Aux/IAA-ARF signalling pathway. qRT-PCR confirmation of the microarray experiment showing the expression of *WRKY23* and genes previously connected to PIN polarity in *HS*::*arx3-1* (D), and in *arf7 arf19* double mutant plants (E). Values represent relative fold change. Error bars indicate standard deviation (see Materials and Methods for detailed description). (F, G) Expression of *WRKY23*::*GUS* in the shoot apical meristem (SAM) and in the presumptive leaf vasculature (G). Besides strong activity in the SAM, GUS staining overlaps with, and partly precedes, the appearance of differentiating vascular strands in young leaves. Two representative plants in consecutive developmental stages are shown. Patchy expression of *WRKY23*::*GUS* in the vasculature of young developing true leaves (G). Arrowheads in F and G depict areas with GUS activity presumably coinciding with future vascular strands that are not morphologically discernible yet.

First, we confirmed and analysed the auxin regulation of *WRKY23* expression. Promoters of auxin-inducible genes typically contain tandem-localized auxin response elements (AuxREs) that are recognised by auxin response factors (ARFs) [47, 48]. ARFs dimerize to act as molecular callipers and provide specificity to the auxin-dependent gene regulation by measuring the distance of AuxREs in the element pair at the promoter [48]. The length of the intergenic region between the 3’-UTR of the previous gene *UPBEAT* (*UPB*; *At2g47270*) and the 5’-UTR of *WRKY23* (*At2g47260*) is 4.5 kbp. The predicted 2.4-kbp *WRKY23* promoter by the AGRIS tool [49] contains 10 AuxRE and AuxRE-like sites and the extended promoter of 3.2 kbp used for native promoter fusion construct [44] contains two additional AuxRE sites (Fig 2A). Such a density of auxin-regulatory sequences in the promoter makes direct regulation by ARF-dependent auxin signalling a plausible scenario.

In accordance with these results, we found that *WRKY23* is auxin inducible in a dose- and time-dependent manner. When we treated *Arabidopsis* seedlings with 100 nM NAA for 4 h, the *WRKY23* transcription increased 2-fold, and 1 μM NAA led to a 6-fold increase (Fig 2B). Time response experiments at the consensus concentration of 10 μM NAA used in PIN lateralization experiments [8] revealed that the *WRKY23* transcription starts to increase approximately after 1.5 h of auxin treatment with a stronger increase after between 2 and 4 h (Fig 2C). This relatively slow auxin-mediated transcriptional regulation of *WRKY23* is well within the time frame for the auxin-mediated PIN lateralization that also occurs strongly only after 4 h [8]. The dependence on the auxin signalling was further supported by the compromised *WRKY23* auxin inducibility in the *HS*::*axr3-1* and *arf7 arf19* mutants (Fig 2D and 2E). These results show that the *WRKY23* transcription depends on the SCF^TIR1^-Aux/IAA-ARF auxin signalling pathway and confirm *WRKY23* as a candidate regulator of auxin-mediated PIN polarization.

A transgenic line harbouring the *uidA* reporter gene (or GUS-coding gene) under the control of a 3.2-kb upstream sequence from *WRKY23* (*WRKY23*::*GUS*), whose expression pattern has previously been confirmed by in situ hybridization [44, 45], revealed that auxin induces the ectopic expression of *WRKY23* in root tissues, partly overlapping with root regions, in which the PIN lateralization can be observed (S1G and S1H Fig). Without auxin treatment, the expression pattern of *WRKY23* partially overlaps with the *DR5* auxin response reporter (S1G and S1I Fig) and auxin distribution as revealed by anti-IAA immunolocalization [44, 45, 50]. Previously, *WRKY23* has been shown to be expressed in all apical cells of an octant stage embryo and at heart stage to be detected in both the root and the shoot stem cell niches (S1D and S1E Fig) [46], possibly indicating that *WRKY23* has—besides its role in root development—also a function in shoot development. We found *WRKY23*::*GUS* expression in pollen grains (S1C Fig), the shoot apical meristem (SAM) (S1A Fig and Fig 2F), as well as at the hydathodes of cotyledons (S1F Fig), coinciding with known auxin response maxima [51]. Sectioning the SAM revealed specific *WRKY23* expression in the L1, L2, and L3 layers (S1A Fig). *WRKY23* promoter activity was prominently associated with the vascular tissues of flowers, cotyledons, and leaves (S1B and S1F Fig and Fig 2G). Notably, the *WRKY23* expression mirrored the pattern of developing leave vasculature with the highest expression in cells adjacent to the differentiated xylem (Fig 2G) and were detected in a venation-like pattern even before any morphological changes typical for the differentiated vasculature were visible (Fig 2F and 2G). In the previous, external auxin source-mediated canalization experiments in pea stems, the PIN channels were preceding the formation of vasculature and later the differentiated xylem formed adjacent to the PIN channels [11]. Thus, the WRKY23 expression pattern in Arabidopsis largely overlaps with presumptive PIN channels being consistent with a role of WRKY23 in venation patterning of leaves–a process regulated by the polarized auxin transport [51, 52].

In summary, the presence of auxin-responsive elements in the promoter, the auxin-inducibility of the *WRKY23* expression together with its dependence on AXR3, ARF7 and ARF19 activities indicate that the *WRKY23* transcription is regulated by Aux/IAA- and ARF-dependent auxin signalling. In addition, the association of the *WRKY23* expression with developing vasculature is consistent with a possible involvement of WRKY23 in the auxin-mediated PIN polarization process.

### WRKY23 gain-of-function leads to PIN1 and PIN2 lateralization

**Fig 3:**
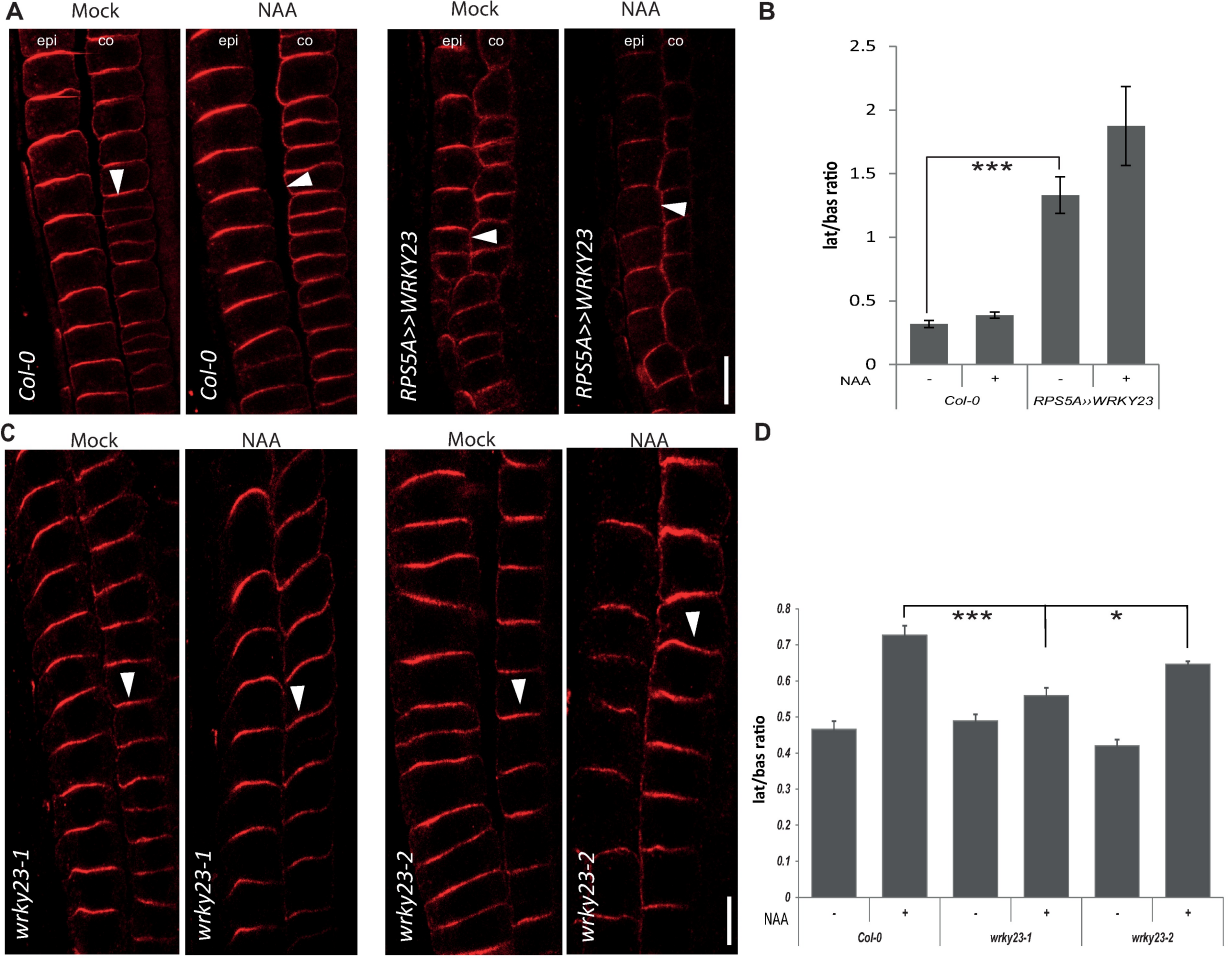
WRKY23 is required for auxin-mediated PIN lateralization in the root. (A) Immunolocalization analysis of PIN2 without or after NAA (4 h, 10 μM) treatment in WT Col-0 and *RPS5A>>WRKY23*. Arrowheads highlight PIN2 polarity. epi, epidermis; co, cortex. (B) Quantitative evaluation of (A) showing mean ratio of PIN2 lateral-to-basal signal intensity in cortex cells. Note that PIN2 lateralization in *RPS5A>>WRKY23* roots is increased even without auxin that still remains effective. Error bars indicate standard error. A One-Way ANOVA test compared marked sets of data (*** *p*<0.0001; *n*>35 cells corresponding to a minimum of 10 roots per treatment and experiment were imaged under comparable conditions). (C) Immunolocalization analysis of PIN2 without or with NAA treatment in WT Col-0 and *wrky23* mutants. Arrowheads highlight representative examples of PIN2 polarity in the. epi, epidermis; co, cortex. (D) Quantitative evaluation of the experiment in (C) showing mean ratio of PIN2 lateral-to-basal signal intensity in endodermal. Error bars indicate standard error. A One-Way ANOVA test compared marked sets of data (* p<0.05; *** *p*<0.001; *n*>100 cells corresponding to a minimum of 10 roots per treatment and experiment were imaged under comparable conditions). Experiments were carried out 3 times; one representative experiment is presented).

Next, we tested whether an altered *WRKY23* expression or activity affected the auxin regulation of the PIN1 and PIN2 protein localization. A strong constitutive overexpression of *WRKY23* was obtained by means of a GAL4-VP16-*UAS* transactivation system (*RPS5A>>WRKY23*) [45, 46, 53]. The *35S* promoter-driven WRKY23 line (*35S*::*WRKY23*) as well as also *35S* promoter-driven dexamethasone-glucocorticoid (DEX/GR) receptor system (*35S*::*WRKY23-GR*) were used for constitutive overexpression, eventually, with inducible nuclear localization [45, 46]. Constitutive overexpression of *WRKY23* had an impact on the PIN2 but not PIN1 polarity. It caused the PIN2 lateralization in root cortex cells, to some extent mimicking the application of auxin (Fig 3A and 3B). Subsequent treatment with NAA further increased lateralization of PIN2 in cortex cells and caused increased lateralization of PIN1 as compared to wild type (Fig 3A and 3B and S2C and S2D Fig). An inducible *WRKY23* gain-of-function line had a similar effect: seedlings of a *35S*::*WRKY23-GR* line treated with DEX to induce WRKY23-GR translocation to the nucleus, resulted in PIN2 but not PIN1 lateralization in the cortex cells. Again, additional NAA treatment had an additive effect on PIN2 lateralization and caused a stronger PIN1 lateralization than as seen in the wild type (S3C and S3D Fig and S2C and S2D Fig).

Thus, both constitutive and inducible *WRKY23* gain-of-function consistently led to PIN2 lateralization and increased the auxin-mediated PIN1 and PIN2 lateralization.

### Repression of WRKY23 activity abolishes the auxin effect on the PIN2 polarization

In complementary experiments, we tested the downregulation effect of the WRKY23 function. The large WRKY family of homologous proteins has an extensive functional redundancy among individual members [54]. As the functional compensation of *wrky23* loss-of-function by other members was likely, given the large size of the *WRKY* gene family, we used a dominant-negative approach with the chimeric repressor silencing technology [55]. This technology is based on a translational fusion of an activating TF with the repressor domain SRDX, thus inhibiting the expression of target genes. The transactivation activity of WRKY23 had previously been verified in a tobacco transient expression assay, in which the activating or repressing potential of the TF fused to GAL4 had been checked in the presence of a *UAS*::*Luciferase* construct [45].

Plants expressing *WRKY23-SRDX* under both the native and constitutive promoters showed a clear auxin insensitivity in PIN2 lateralization, namely the auxin treatment did not lead to lateralization when compared to the controls (S3A and S3B Fig). Notably, PIN1 lateralization did not change visibly after NAA treatment (S2C and S2D Fig).

### *wrky23* partial loss-of-function mutants are defective in auxin impact on the PIN polarity

**Fig 4:**
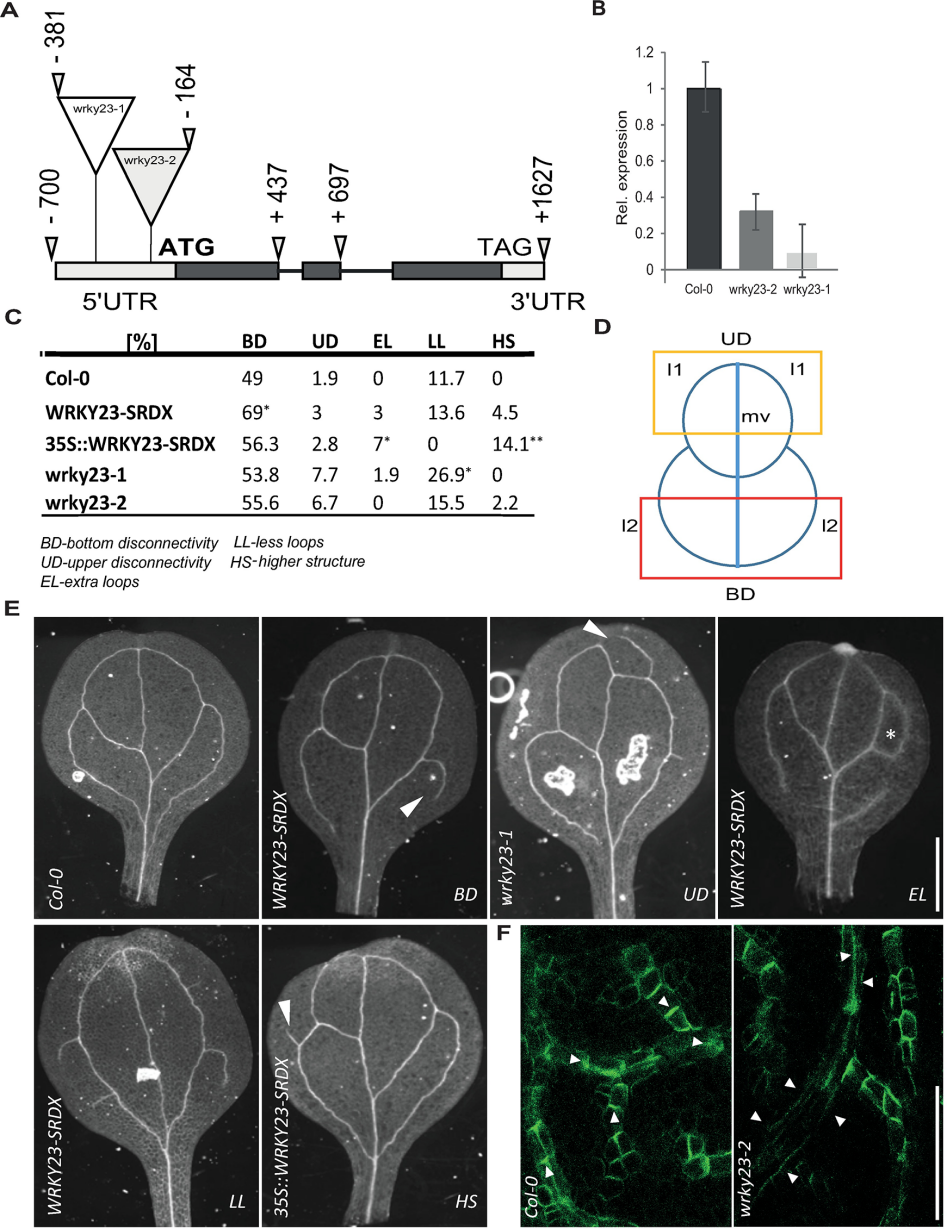
Isolation and characterization of *wrky23* mutants. (A) Schematic representation of the *WRKY23* locus. Exons are represented by boxes, while introns are shown as lines. Coding regions are filled with dark grey. Exact locations of the T-DNA insertions are depicted. (B) qRT-PCR analysis of *WRKY23* expression in the isolated mutant lines. Relative expression values are normalized to the level detected in WT Col-0. See Materials and Methods for more details. (C) Evaluation of cotyledon vasculature defects in *WRKY23-SRDX*, *35S*::*WRKY23-SRDX* and *wrky23* mutants. A One-Way ANOVA test compared marked sets of data (* p<0.05; *** *p*<0.001; *n*>50 cotyledons). (D) Schematic representation of cotyledon vasculature pattern. l1, first loop; l2, second loop; mv, midvein. Yellow and red box delineate UD and BD zone of evaluating. (E) Representative images of analysed vasculature defects. (F) Representative images of immunolocalization analysis of PIN1 in developing young first leaves. In the WT, PIN1 shows typical polarization, whereas in *wrky23-2* mutant this polarization is abolished. At least 50 leaves per genotype were analysed.

To investigate intrafamily redundancy and to assess specifically the role of WRKY23 on the auxin effect on the PIN polarity, we isolated two T-DNA insertional mutants in the *WRKY23* locus, designated *wrky23-1* and *wrky23-2* (Fig 4A). The quantitative reverse transcription-polymerase chain reaction (qRT-PCR) analysis revealed that both alleles are knock-downs, *wrky23-1* having more downregulated expression (Fig 4B).

Similarly to the *WRKY23-SRDX* lines, both *wrky23* mutant alleles showed a reduced PIN2 lateralization response to auxin treatment and, additionally, also reduced PIN1 lateralization. Specifically, following the NAA treatment, the PIN1 and PIN2 lateralization in root endodermal cells was diminished in the *wrky23-2* weaker knock-down and, even more so, in the stronger *wrky23-1* allele (S2A and S2B Fig and Fig 3C and 3D). The observed opposite effects of WRKY23 gain- and loss-of-function on the PIN lateralization suggested that WRKY23 plays an important role in the auxin-mediated PIN polarity rearrangements.

### WRKY23 plays a role in PIN polarization during venation patterning

The importance of a tight PIN polarity regulation for directional auxin fluxes and plant growth and development has been demonstrated previously [2, 3]. Therefore, we analysed the phenotypes related to PIN polarity or auxin transport in transgenic lines with an altered expression or activity of *WRKY23*. *35S*::*WRKY23* overexpressing plants show growth retardation and root meristem patterning defects [45]. Also, dominant negative lines showed severe defects in lateral root organogenesis [45]. Both *WRKY23-SRDX* and *35S*::*WRKY23* lines had shorter roots than those of Col-0 (S4A Fig) and *WRKY23-SRDX* showed defects in gravitropism, similar to those observed in the auxin transport mutant *pin2/eir1* [56, 57]. Notably, native promoter-driven *WRKY23-SRDX* displayed a significant increase in lateral root density (S4B Fig). Notably, none of these phenotypical defects, including root meristem disorganization, root growth inhibition, and lateral root development alteration, were observed in the *wrky23* mutant alleles (S4A and S4B Fig), suggesting that these more pleiotropic defects are not related to the WRKY23 action specifically, but they could reflect a broader role of the *WRKY* gene family in plant development.

The canalization hypothesis proposed that the leaf venation pattern depends on the auxin feedback on the PIN polarity [58]. We analysed several features of vascular defects in cotyledons.–bottom disconnectivity of l2 vein loops (BD), upper disconnectivity of l1 vein loops (UD), extra loops (EL), less loops (LL) and appearance of higher order structures (HS) (Fig 4C–4E). In plants expressing *WRKY23*::*WRKY23-SRDX* and *35S*::*WRKY23-SRDX*, we observed vasculature patterning defects manifested by increased incidence in BD, HS and EL On the other hand, both *wrky23-1* and *wrky23-2* mutant alleles showed more defects in UD and LL (Fig 4C).

Next, we tested the PIN1 polarity during vascular tissue development by means of anti-PIN1 antibody staining on young first leaves. In the WT leaves, the staining revealed a pronounced PIN1 polarization along the basipetal (rootward) direction (S4C Fig). In the *35S*::*WRKY23* and *WRKY23-SRDX* lines, the typical PIN1 polarity was partly or completely abolished in some veins or their parts (S4C Fig). Similar PIN1 polarity defects were also found in *wrky23-1* and *wrky23-2* lines (Fig 4F and S4C Fig). The venation defects might be interpreted in terms of defective canalization (as suggested by the PIN1 polarity defects), although the venation defects differ somewhat from defects induced by auxin transport inhibition [51, 52]. This observation indicates that interference with the PIN polarization does not have the same consequence as inhibition of PIN auxin transport activity.

In summary, our genetic analysis revealed that from the numerous functions of the WRKY family in the regulation of plant development [45, 46], WRKY23 is more specifically involved in auxin-mediated PIN polarity rearrangements and leave venation patterning.

## Discussion

Classical experiments have led to the formulation of the so-called canalization hypothesis that proposes an auxin feedback on the auxin transport and consequent formation of auxin channels as a central element of multiple self-organizing developmental processes; in particular formation and regeneration of vasculature [7]. In canalization, the auxin transport through an initially homogeneous tissue follows a self-organizing pattern, leading from initially broad fields of auxin-transporting cells to eventually a narrow transport channel, consequently establishing the position of future vascular veins [6]. This hypothesis [4, 5] is further supported by successful modelling efforts based on the concerted cellular polarization via a feedback mechanism, by which auxin influences the directionality of its own flow by polarity rearrangement of auxin carriers [6, 15, 59–62]. Most of these models rely on hypothetical propositions, such as auxin flux sensors or direct cell-to-cell communication, giving testimony of our lack of understanding how canalization works mechanistically. However, the auxin impact on the PIN polarization has been experimentally demonstrated in different contexts and this effect has been shown to rely on the transcriptional gene expression activation through auxin signalling [8, 9, 11, 19].

Our transcriptional profiling experiments on auxin-dependent PIN rearrangements in *Arabidopsis* roots provide insight into the transcriptional reprogramming during auxin-mediated PIN polarity rearrangements and identify potential downstream molecular components in this process, including established PIN polarity regulators, such as PID, PIP5K, and PATELLINS [28, 30, 37, 63], validating the soundness of the experimental concept. Among a number of novel components awaiting further characterization, we also found the transcriptional activator *WRKY23*.

*WRKY23* is an auxin-responsive gene. The local upregulation of the *WRKY23* expression following the auxin application is consistent with a possible involvement in the PIN repolarization process. The *WRKY23* transcription is induced by auxin in a dose- and time-dependent manner and it is reminiscent of the expression pattern of the *DR5rev* auxin signalling reporter. Notably, *WRKY* genes are traditionally known to be involved in defensive processes in plants. More and more, this limited functional spectrum has been broadened by studies uncovering the involvement of these TFs in developmental and physiological processes other than plant defense [45, 46, 64, 65]. In the case of *WRKY23*, besides a role in plant-nematode interaction with subsequent activation of auxin responses, participation in auxin transport through flavonol synthesis in the root as well as a function in a *mp/bdl-*dependent pathway in embryo development have been demonstrated [44–46].

We show that *WRKY23* is a crucial factor required for auxin-mediated PIN polarity rearrangements, because gain-of-function and dominant-negative *WRKY23* lines as well as *wrky23* mutants were strongly affected in this process. These defects at the cellular level revealed by the exogenous auxin application appears to be developmentally relevant, because *wrky23* mutants are defective also in the PIN1 polarization process during vascular tissue formation of leaf venation and consequently in vascular tissue formation. Notably, increased PIN2 but not PIN1 lateralization in the *WRKY23* overexpression lines and PIN2 but not PIN1 insensitivity to auxin treatment in *WRKY23-SRDX* lines indicate a partly diverging mechanism controlling PIN1 and PIN2 relocation. This is consistent with reported differences in PIN1 and PIN2 trafficking mechanisms [66].

Our results also suggest that *WRKY23* is a critical player in auxin feedback on PIN polar localization. As a TF, *WRKY23* is probably not directly involved in regulating localization of transmembrane proteins, such as PIN proteins. Instead, this work opens avenues for future studies revealing the *WRKY23*-dependent transcriptional network. The identification of WRKY23 and its role in the auxin feedback on the PIN polarity along with other established PIN polarity regulators proves that our transcriptomics dataset can be mined in the future to identify additional regulators. Ultimately, it will provide insights into the molecular mechanism of this key aspect of the canalization-dependent regulation of plant development.

## Materials and methods

### Plant material and growth conditions

All *Arabidopsis thaliana* (L.) Heynh. lines were in Columbia-0 (Col-0) background. The insertional mutants *wrky23-1* (SALK_003943) and *wrky23-2* (SALK_38289) were obtained from NASC and genotyped with the primers listed in S3 Table. The *arf7 arf19* double mutant and the *HS*::*axr3-1* transgenic line have been described previously [26, 29] as well as the *DR5*::*GUS* [18] and *PIN1-GFP* [67]. For *RPS5A>>WRKY23* analyses, the F1 generation of a *RPS5A*::*GAL4VP16* [53] × *UAS*::*WRKY23* [45] cross was analysed and compared with the F1 generations from the *UAS*::*WRKY23* × WT Col-0 and *RPS5A*::*GAL4VP16* × WT Col-0 crosses. *WRKY23*::*GUS*, *35S*::*WRKY23-GR*,*35S*::*WRKY23*, *WRKY23*::*WRKY23-SRDX*, and *35S*::*WRKY23-SRDX* have been described previously [44, 45]. Seeds were surface-sterilized overnight by chlorine gas, sown on solid *Arabidopsis* medium (AM+; half-strength MS basal salts, 1% [w/v] sucrose, and 0.8% [w/v] phytoagar, pH 5.7), and stratified at 4°C for at least 2 days prior to transfer to a growth room with a 16-h light/8-h dark regime at 21°C. The seedlings were grown vertically for 4 or 6 days, depending on the assay.

*Arabidopsis* seedlings were treated with auxin or chemicals in liquid AM+ at 21°C in a growth room with the following concentrations and times: for α-naphthaleneacetic acid (NAA; Sigma-Aldrich) at 10 μM for 4 h; dexamethasone (DEX; Sigma-Aldrich) 10 μM for 24 h. Mock treatments were done with equivalent amounts of DMSO.

### Microarray analysis

Wild type Col-0 and *HS*::*axr3-1* seeds were grown vertically on AM+ plates for 5 days. We applied a 40 min heat shock at 37°C to the seedlings, followed by a 1.5-h recovery at normal growth temperature. Subsequently, the seedlings were transferred to liquid AM+ and treated with 10 μM NAA or DMSO for 4 h. Afterward, the lower third of 100–130 roots from each treatment was cut off, frozen in liquid N_2_. RNA was extracted with the RNAeasy mini kit (Qiagen). Probes were prepared and hybridized to the *Arabidopsis* ATH1–121501 gene expression array (Affymetrix) as described [68]. Expression data for Col-0, *HS*::*axr3-1*, both NAA and mock treated, had been deposited under the ArrayExpress number E-MEXP-3283. Expression profiling data for *arf7 arf19* (ArrayExpress: E-GEOD-627) have been published previously [29]. Raw data were pairwise analyzed with the logit-t algorithm [69] with a cutoff of *p* = 0.05.

### RNA extraction, cDNA synthesis, and quantitative RT-PCR and analysis

RNA extraction, cDNA synthesis, and quantitative (q)RT-PCR were done as described [37]. Selected candidate gene transcript levels were quantified with qRT-PCR with specific primer pairs, designed with Primer-BLAST (http://www.ncbi.nlm.nih.gov/tools/primer-blast/). Transcript levels were normalized to *GAMMA-TUBULIN 2* (*TUB2*; *AT5G05620*), which was constitutively expressed and auxin independent across samples. All PCRs were run in three biological replicates per three technical repeats. The data were processed with a qRT-PCR analysis software (Frederik Coppens, Ghent University-VIB, Ghent, Belgium). Primers used in this study are listed in the S3 Table.

### Whole-mount *in situ* immunolocalization, microscopy, and quantitative PIN relocalization analysis

PIN immunolocalizations of primary roots and young leaves were carried out as described [70]. The antibodies were used as follows: anti-PIN1, 1:1000 [13] and anti-PIN2, 1:1000 [71]. For primary roots, the secondary goat anti-rabbit antibody coupled to Cy3 (Sigma-Aldrich) was diluted 1:600. For young leaves, the secondary goat anti-rabbit antibody coupled to Alexa Fluor 488 (Sigma-Aldrich) was diluted 1:600. For confocal microscopy, a Zeiss LSM 700 confocal microscope was used. The PIN relocalization was quantitative analysed as described [8], at least 3 experiments were performed for each observation. Note that the absolute levels of the PIN lateralization index may vary between individual experiments (depending on the anti-PIN signal strength), but the relative differences are always consistent.

### Phenotypic analysis

All measurements were done with ImageJ (http://rsb.info.nih.gov/ij). For the root length analysis 6-day-old seedlings were scanned and root lengths were measured. For the lateral roots analysis 10-day-old seedlings were scanned and lateral root density was calculated from ratio number of LR/root length.

### Histological analyses and microscopy

To detect β-glucuronidase (GUS) activity, seedlings were incubated in reaction buffer containing 0.1 M sodium phosphate buffer (pH 7), 1 mM ferricyanide, 1 mM ferrocyanide, 0.1% Triton X-100, and 1 mg/ml X-Gluc for 2 h in the dark at 37°C. Afterward, chlorophyll was removed by destaining in 70% ethanol and seedlings were cleared.

Tissues (seedlings and cotyledons) were cleared in a solution containing 4% HCl and 20% methanol for 15 min at 65°C, followed by a 15-min incubation in 7% NaOH and 70% ethanol at room temperature. Next, seedlings were rehydrated by successive incubations in 70%, 50%, 25%, and 10% ethanol for 5 min, followed by incubation in a solution containing 25% glycerol and 5% ethanol. Finally, seedlings were mounted in 50% glycerol and monitored by differential interference contrast microscopy DIC (Olympus BX53) or a stereomicroscope (Olympus SZX16).

## Supporting information

S1 Fig Pattern of *GUS* expression in *WRKY23*::*GUS* plants. (A) SAM section showing specific *WRKY23* expression in the L1, L2, and L3 layers. (B) *WRKY23* expression in the pistil vasculature. (C) Anther showing *WRKY23*::*GUS* activity in pollen (inset). (D) GUS staining of *WRKY23*::*GUS* embryos showing promoter activity in all apical cells of an early globular embryo. (E) GUS activity in the SAM and RAM of an early torpedo stage embryo. (F) Cotyledon showing GUS staining at the hydathode (h) and in the vasculature. (G-J) *WRKY23* promoter activation by auxin treatment. G and H: Expression pattern of *WRKY23*::*GUS* in the root changes following 6 h of auxin treatment. GUS staining becomes generally stronger and additionally expressed in the meristematic and transition zones of the root tip/arrowhead). I and J: *DR5*::*GUS* activity under the same experimental conditions as in (G-H).
(PDF)

S2 Fig Polarity of PIN1 in *WRKY23* transgenic lines. (A and B) Immunolocalization of PIN1 in *wrky23* mutants and *arf7/19* lines revealing reduced lateralization of PIN1. Arrowheads highlight PIN1 polarity. en, endodermis; per, pericycle. Graph shows mean ratio of lateral-to-basal signal intensity of PIN1 in endodermal cells. Error bars indicate standard error. A One-Way ANOVA test compared marked sets of data (*** *p*<0.0001; *n*>60 cells corresponding to a minimum of 10 roots per treatment and per experiment imaged under comparable conditions). Experiments were carried out at least 3 times; one representative experiment is shown. (C) Immunolocalization of PIN1 in dominant-negative *WRKY23-SRDX* plants driven by native promoter and overexpression lines - *35S*::*WRKY23*, *35S*::*WRKY23-GR*. WT Col-0 was used as a control. Arrowheads highlight PIN1 polarity in endodermal cells. en, endodermis; per, pericycle. Bar = 10 μm. (D) Quantitative evaluation of (C) showing mean ratio of lateral-to-basal signal intensity of PIN1 in cortex cells. Error bars indicate standard error. A One-Way ANOVA test compared marked sets of data (*** *p*<0.0001; *n*>60 cells corresponding to a minimum of 10 roots per treatment and per experiment were imaged under comparable conditions). Experiments were carried out at least 3 times; one representative experiment is shown.
(PDF)

S3 Fig Polarity of PIN2 in *WRKY23* transgenic lines. (A) Immunolocalization of PIN2 in dominant-negative *WRKY23-SRDX* plants driven by native and constitutive promoter. WT Col-0 was used as a control (see Fig 3A and quantification in S3B). Arrowheads highlight PIN2 polarity in cortex cells. epi, epidermis; co, cortex. Bar = 10 μm. (B) Quantitative evaluation of (A) showing mean ratio of lateral-to-basal signal intensity of PIN2 in cortex cells. Error bars indicate standard error. A One-Way ANOVA test compared marked sets of data (*** *p*<0.0001; *n*>70 cells corresponding to a minimum of 10 roots per treatment and per experiment were imaged under comparable conditions). Experiments were carried out at least 3 times; one representative experiment is shown.
(C) Immunolocalization of PIN2 in DEX-inducible *35S*::*WRKY23-GR* plants treated with DEX and/or NAA. WT Col-0 was used as control (see quantification in S3D). Arrowheads highlight PIN2 polarity in cortex cells. epi, epidermis; co, cortex. Bar = 10 μm. (D) Graph showing mean ratio of lateral-to-basal signal intensity of PIN2 in cortex cells. Induced *35S*::*WRKY23-GR* roots show slightly more PIN2 lateralization without auxin that is apparently more effective to increase PIN2 lateralization in this line than the controls. Error bars indicate standard error. A One-Way ANOVA test compared marked sets of data (*** *p*<0.0001, * *p*<0.05; *n*>35 cells corresponding to a minimum of 10 roots per treatment and per experiment were imaged under comparable conditions). Experiments were carried out at least 3 times; one representative experiment is shown.
(PDF)

S4 Fig Phenotype defects in WRKY23 transgenic lines and *wrky23* mutants. (A) Primary root length of 6-day-old transgenic lines and *wrky23* mutants. Central lines show median values; box limits indicate the 25^th^ and 75^th^ percentiles as determined by the R software; whiskers extend 1.5 times the interquartile range from the 25^th^ and 75^th^ percentiles. Significance was determined by two-tailed equal T-test between Col-0 and other lines; (*** *p*<0.001); n>60 roots per line. (B) Lateral root density in plants with impaired WRKY23 function. WRKY-SRDX denotes *WRKY23*::*WRKY23-SRDX*.Box plot properties and statistical analysis are as in (A). n>80 roots per line. (C) Immunolocalization analysis of PIN1 in developing true leaves. In the WT, PIN1 shows typical polarization towards the leaf base, whereas in WRKY23 transgenic lines and *wrky23* mutants this polarization of some branches is abolished. Arrowheads highlights defective PIN1 polarization in vasculature. At least 50 leaves per genotype were analysed. (D) Quantitative evaluation of (C) showing percentage of abolished PIN1 polarity. At least 50 branches per genotype were analysed.
(PDF)

S1 Table Candidate genes from the microarray experiment. (A) Venn diagram representing gene overlay of microarray experiments. Dataset of auxin-regulated genes in WT Col-0 seedlings was overlaid with a second set of genes acquired from the comparison of auxin-treated WT Col-0 and heat-shock—induced auxin-treated *HS*::*axr3-1* lines. Overlap of these genes yielded a list of 245. (B) List of the 245 genes. Gene model descriptions are depicted as they appear in the TAIR database.
(PDF)

S2 Table Narrowed-down list of candidate genes from the microarray experiments. (A) Venn diagram representing gene overlay of microarray experiments. Datasets of genes differentially regulated in *HS*::*axr3-1* compared to auxin-regulated genes in WT Col-0 were overlaid with a third set of genes that are no longer auxin regulated in the *arf7 arf19* background [29]. Overlap of all three microarrays gave 125 genes. (B) List of the 125 overlapping genes containing putative polarity regulators. Gene model descriptions are depicted as they appear in the TAIR database.
(PDF)

S3 Table List of PCR primers used. (PDF)
