# Supplementary figures and images for: WRKY23 is a component of the transcriptional network mediating auxin feedback on PIN polarity
Source: PLoS Genet. 2018 Jan 29;14(1):e1007177. doi: 10.1371/journal.pgen.1007177 (PMC5805370; doi:10.1371/journal.pgen.1007177)

S1 Figure

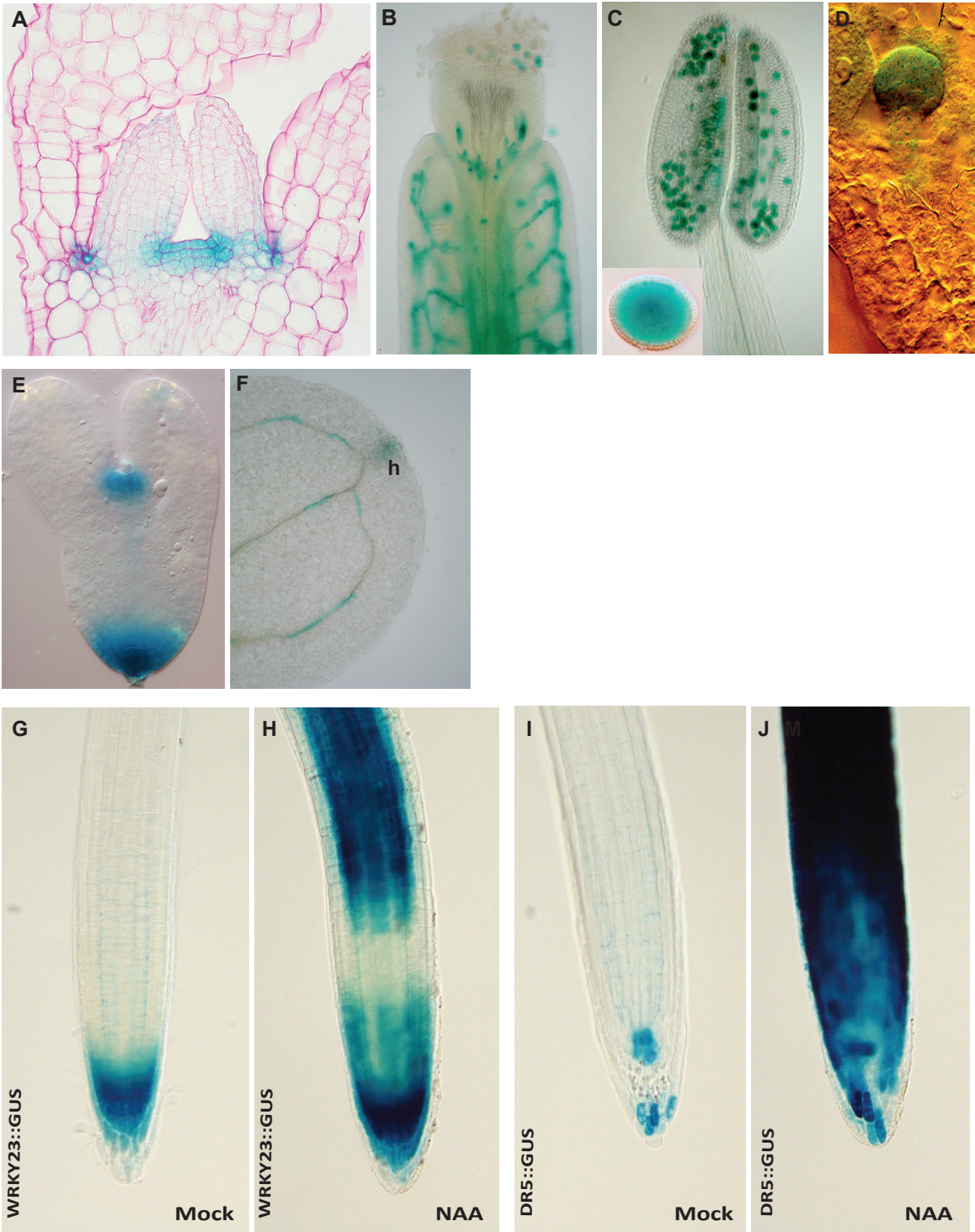

Supplement: S1 Fig — (A) SAM section showing specific WRKY23 expression in the L1, L2, and L3 layers. (B) WRKY23 expression in the pistil vasculature. (C) Anther showing WRKY23::GUS activity in pollen (inset). (D) GUS staining of WRKY23::GUS embryos showing promoter activity in all apical cells of an early globular embryo. (E) GUS activity in the SAM and RAM of an early torpedo stage embryo. (F) Cotyledon showing GUS staining at the hydathode (h) and in the vasculature. (G-J) WRKY23 promoter activation by auxin treatment. G and H: Expression pattern of WRKY23::GUS in the root changes following 6 h of auxin treatment. GUS staining becomes generally stronger and additionally expressed in the meristematic and transition zones of the root tip/arrowhead). I and J: DR5::GUS activity under the same experimental conditions as in (G-H). (PDF) [file pgen.1007177.s001.pdf]

S2 Figure

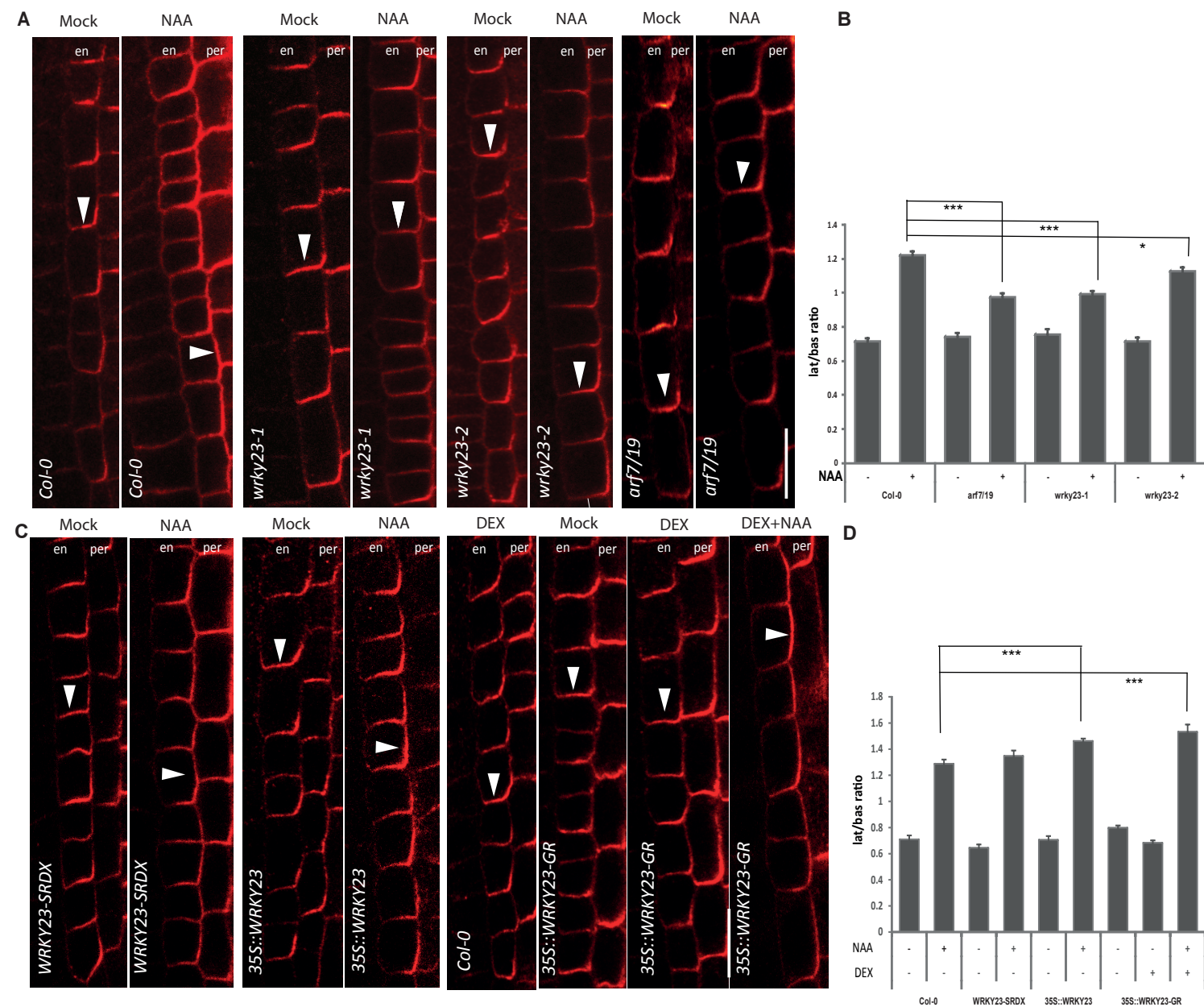

Supplement: S2 Fig — (A and B) Immunolocalization of PIN1 in wrky23 mutants and arf7/19 lines revealing reduced lateralization of PIN1. Arrowheads highlight PIN1 polarity. en, endodermis; per, pericycle. Graph shows mean ratio of lateral-to-basal signal intensity of PIN1 in endodermal cells. Error bars indicate standard error. A One-Way ANOVA test compared marked sets of data (*** p<0.0001; n>60 cells corresponding to a minimum of 10 roots per treatment and per experiment imaged under comparable conditions). Experiments were carried out at least 3 times; one representative experiment is shown. (C) Immunolocalization of PIN1 in dominant-negative WRKY23-SRDX plants driven by native promoter and overexpression lines - 35S::WRKY23, 35S::WRKY23-GR. WT Col-0 was used as a control. Arrowheads highlight PIN1 polarity in endodermal cells. en, endodermis; per, pericycle. Bar = 10 μm. (D) Quantitative evaluation of (C) showing mean ratio of lateral-to-basal signal intensity of PIN1 in cortex cells. Error bars indicate standard error. A One-Way ANOVA test compared marked sets of data (*** p<0.0001; n>60 cells corresponding to a minimum of 10 roots per treatment and per experiment were imaged under comparable conditions). Experiments were carried out at least 3 times; one representative experiment is shown. (PDF) [file pgen.1007177.s002.pdf]

S3 Figure

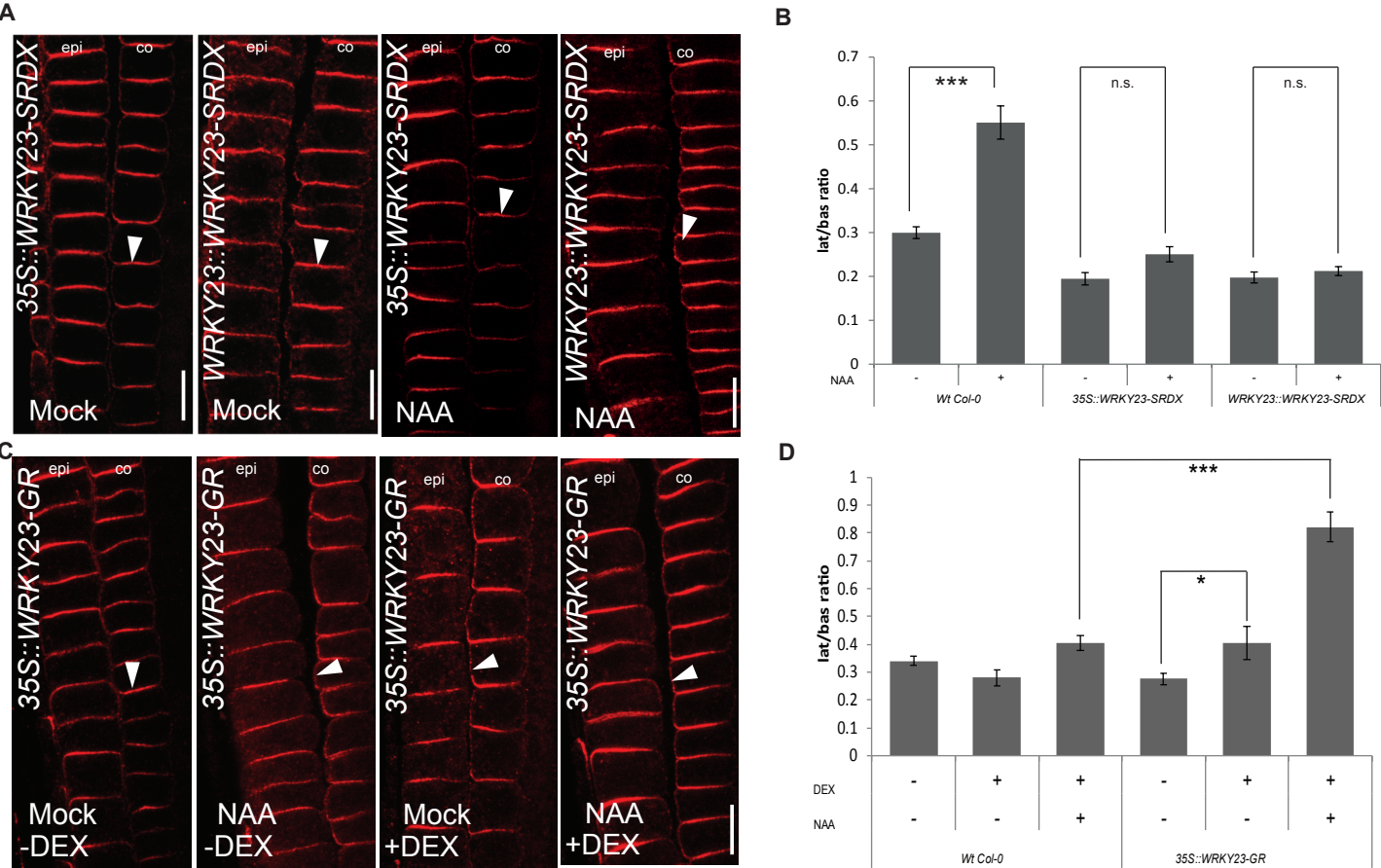

Supplement: S3 Fig — (A) Immunolocalization of PIN2 in dominant-negative WRKY23-SRDX plants driven by native and constitutive promoter. WT Col-0 was used as a control (see Fig 3A and quantification in S3B). Arrowheads highlight PIN2 polarity in cortex cells. epi, epidermis; co, cortex. Bar = 10 μm. (B) Quantitative evaluation of (A) showing mean ratio of lateral-to-basal signal intensity of PIN2 in cortex cells. Error bars indicate standard error. A One-Way ANOVA test compared marked sets of data (*** p<0.0001; n>70 cells corresponding to a minimum of 10 roots per treatment and per experiment were imaged under comparable conditions). Experiments were carried out at least 3 times; one representative experiment is shown. (C) Immunolocalization of PIN2 in DEX-inducible 35S::WRKY23-GR plants treated with DEX and/or NAA. WT Col-0 was used as control (see quantification in S3D). Arrowheads highlight PIN2 polarity in cortex cells. epi, epidermis; co, cortex. Bar = 10 μm. (D) Graph showing mean ratio of lateral-to-basal signal intensity of PIN2 in cortex cells. Induced 35S::WRKY23-GR roots show slightly more PIN2 lateralization without auxin that is apparently more effective to increase PIN2 lateralization in this line than the controls. Error bars indicate standard error. A One-Way ANOVA test compared marked sets of data (*** p<0.0001, * p<0.05; n>35 cells corresponding to a minimum of 10 roots per treatment and per experiment were imaged under comparable conditions). Experiments were carried out at least 3 times; one representative experiment is shown. (PDF) [file pgen.1007177.s003.pdf]

**S4 Figure**

**A**

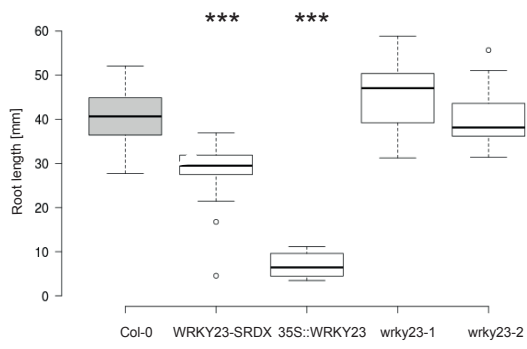

**B**

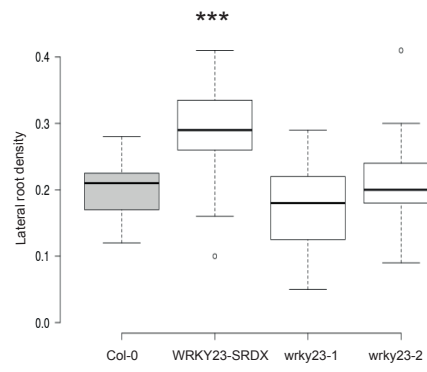

**C**

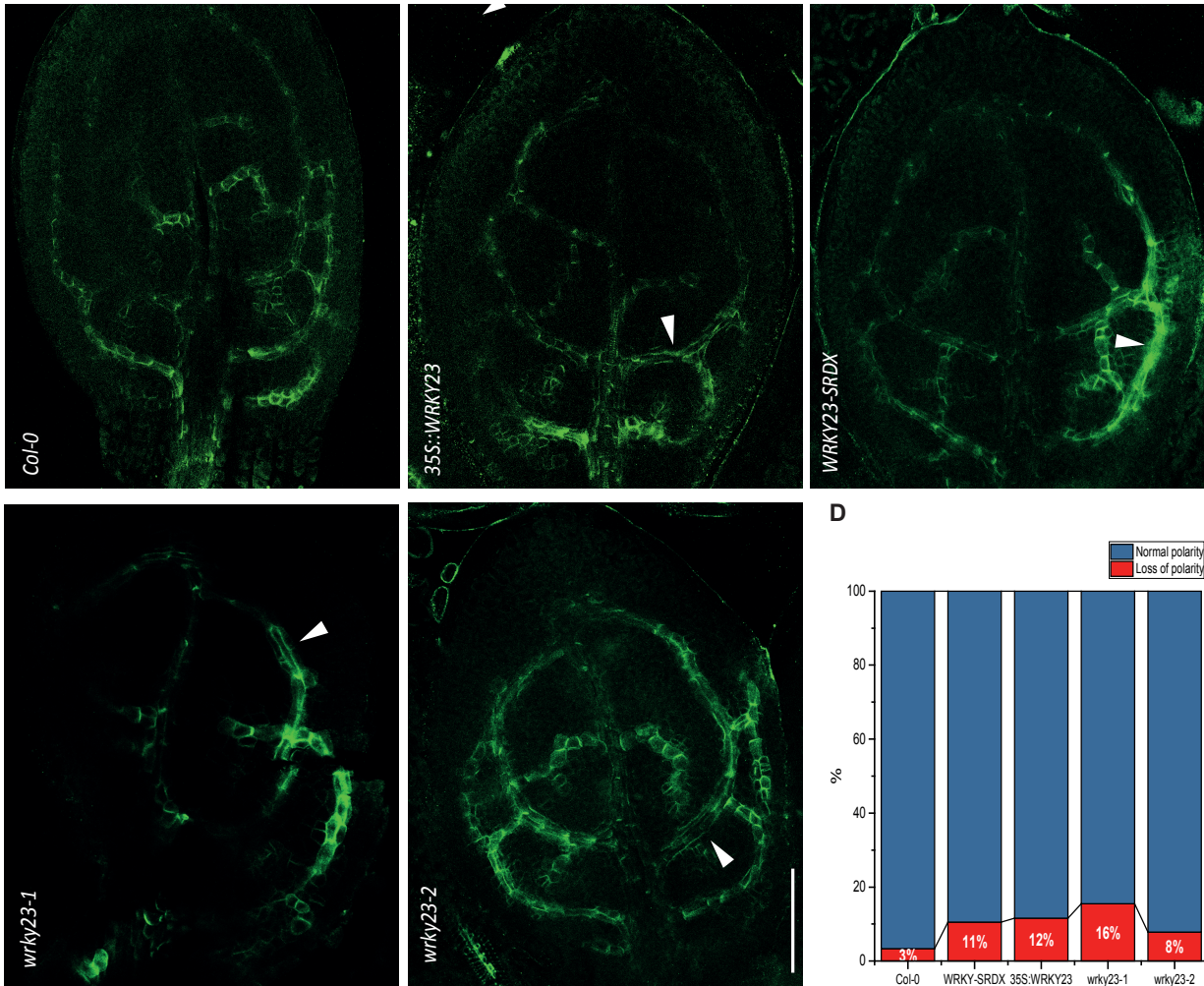

**D**

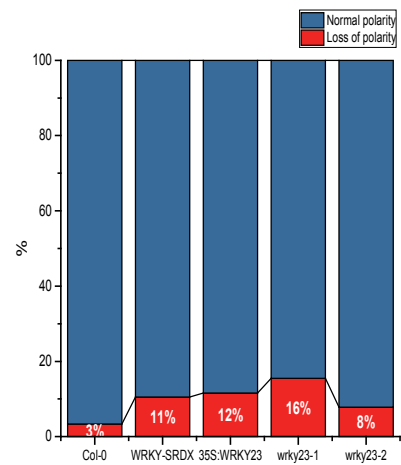

Supplement: S4 Fig — (A) Primary root length of 6-day-old transgenic lines and wrky23 mutants. Central lines show median values; box limits indicate the 25th and 75th percentiles as determined by the R software; whiskers extend 1.5 times the interquartile range from the 25th and 75th percentiles. Significance was determined by two-tailed equal T-test between Col-0 and other lines; (*** p<0.001); n>60 roots per line. (B) Lateral root density in plants with impaired WRKY23 function. WRKY-SRDX denotes WRKY23::WRKY23-SRDX.Box plot properties and statistical analysis are as in (A). n>80 roots per line. (C) Immunolocalization analysis of PIN1 in developing true leaves. In the WT, PIN1 shows typical polarization towards the leaf base, whereas in WRKY23 transgenic lines and wrky23 mutants this polarization of some branches is abolished. Arrowheads highlights defective PIN1 polarization in vasculature. At least 50 leaves per genotype were analysed. (D) Quantitative evaluation of (C) showing percentage of abolished PIN1 polarity. At least 50 branches per genotype were analysed. (PDF) [file pgen.1007177.s004.pdf]
