# Supplementary material for: WRKY23 is a component of the transcriptional network mediating auxin feedback on PIN polarity
Source: PLoS Genet. 2018 Jan 29;14(1):e1007177. doi: 10.1371/journal.pgen.1007177 (PMC5805370; doi:10.1371/journal.pgen.1007177)
Supplement: S2 Table — (A) Venn diagram representing gene overlay of microarray experiments. Datasets of genes differentially regulated in HS::axr3-1 compared to auxin-regulated genes in WT Col-0 were overlaid with a third set of genes that are no longer auxin regulated in the arf7 arf19 background [29]. Overlap of all three microarrays gave 125 genes. (B) List of the 125 overlapping genes containing putative polarity regulators. Gene model descriptions are depicted as they appear in the TAIR database. (PDF) [file pgen.1007177.s006.pdf]

S2 Table

A

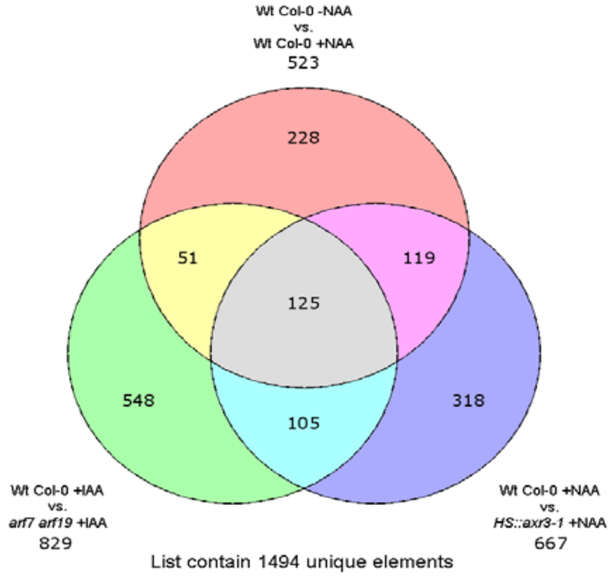

B

Locus identifier	Primary Gene Symbol	Gene Model Description
AT1G02400	GIBBERELLIN 2-OXIDASE 6 (GA2OX6)	Encodes a gibberellin 2-oxidase that acts on C19 gibberellins but not C20 gibberellins.
AT1G03870	FASCICLIN-LIKEARABINOOGALACTAN 9 (FLA9)	fasciclin-like arabinogalactan-protein 9 (Fla9)
AT1G08500	EARLY NODULIN-LIKEPROTEIN 18 (ENODL18)	early nodulin-like protein 18 (ENODL18); FUNCTIONS IN: electron carrier activity, copper ion binding; LOCATED IN: anchored to membrane;
AT1G15580	INDOLE-3-ACETIC ACIDINDUCIBLE 5 (IAA5)	auxin induced protein
AT1G19220	AUXIN RESPONSE FACTOR19 (ARF19)	Encodes an auxin response factor that contains the conserved VP1-B3 DNA-binding domain at its N-terminus and the Aux/IAA-like domains III and IV present in most ARFs at its C-terminus. The protein interacts with IAA1 (yeast two hybrid) and other auxin response elements such as ER7 and ER9 (yeast one hybrid). ARF19 protein can complement many aspects of the arf7 mutant phenotype and , together with ARF7, is involved in the response to ethylene. In the arf7 arf19 double mutant, several auxin- responsive genes (e.g. IAA5, LBD16, LBD29 and LBD33) are no longer upregulated by auxin.
**AT1G21980**	**PHOSPHATIDYLINOSITO L-4-PHOSPHATE 5- KINASE 1 (PIP5K1)**	**Type I phosphatidylinositol-4-phosphate 5-kinase. Preferentially phosphorylates PtdIns4P. Induced by water stress and abscisic acid in Arabidopsis thaliana. Expressed in procambial cells of leaves, flowers and roots. A N-terminal Membrane Occupation and Recognition Nexus (MORN) affects enzyme activity and distribution.**
AT1G23080	PIN-FORMED 7 (PIN7)	Encodes a novel component of auxin efflux that is located apically in the basal cell and is involved during embryogenesis in setting up the apical-basal axis in the embryo. It is also involved in pattern specification during root development. In roots, it is expressed at lateral and basal membranes of provascular cells in the meristem and elongation zone, whereas in the columella cells it coincides with the PIN3 domain. Plasma membrane-localized PIN proteins mediate a saturable efflux of auxin. PINs mediate auxin efflux from mammalian and yeast cells without needing additional plant-specific factors. The action of PINs in auxin efflux is distinct from PGPs, rate-limiting, specific to auxins and sensitive to auxin transport inhibitors. PINs are directly involved of in catalyzing cellular auxin efflux.
AT1G28370	ERF DOMAIN PROTEIN 11 (ERF11)	encodes a member of the ERF (ethylene response factor) subfamily B-1 of ERF/AP2 transcription factor family. The protein contains one AP2 domain. There are 15 members in this subfamily including ATERF-3, ATERF-4, ATERF-7, and leafy petiole.
AT1G28680		HXXXD-type acyl-transferase family protein
AT1G29500		SAUR-like auxin-responsive protein family
AT1G29510	SMALL AUXIN UPREGULATED 68(SAUR68)	SMALL AUXIN UPREGULATED 68 (SAUR68);
AT1G33790		jacalin lectin family protein;
AT1G50660		unknown protein;
AT1G52830	INDOLE-3-ACETIC ACID 6 (IAA6)	An extragenic dominant suppressor of the hy2 mutant phenotype. Also exhibits aspects of constitutive photomorphogenetic phenotype in the absence of hy2. Mutants have dominant leaf curling phenotype shortened hypocotyls and reduced apical hook. Induced by indole-3-acetic acid.
AT1G60010		unknown protein
AT1G62770		Plant invertase/pectin methylesterase inhibitor superfamily protein
AT1G64405		unknown protein
AT1G69530	EXPANSIN A1 (EXPA1)	Member of Alpha-Expansin Gene Family. Naming convention from the Expansin Working Group (Kende et al, Plant Mol Bio). Involved in the formation of nematode-induced syncytia in roots of Arabidopsis thaliana.
AT1G70560	TRYPTOPHAN AMINOTRANSFERASE OF ARABIDOPSIS 1 (TAA1)	TAA1 is involved in the shade-induced production of indole-3-pyruvate (IPA), a precursor to IAA, a biologically active auxin. It is also involved in regulating many aspects of plant growth and development from embryogenesis to flower formation and plays a role in ethylene-mediated signaling. This enzyme can catalyze the formation of IPA from L-tryptophan. Though L-Trp is expected to be the preferred substrate in vivo, TAA1 also acts as an aminotransferase using L-Phe, L-Tyr, L-Leu, L-Ala, L-Met, and L-Gln.
AT1G70940	PIN-FORMED 3 (PIN3)	A regulator of auxin efflux and involved in differential growth. PIN3 is expressed in gravity-sensing tissues, with PIN3 protein accumulating predominantly at the lateral cell surface. PIN3 localizes to the plasma membrane and to vesicles. In roots, PIN3 is expressed without pronounced polarity in tiers two and three of the columella cells, at the basal side of vascular cells, and to the lateral side of pericycle cells of the elongation zone. PIN3 overexpression inhibits root cell growth. Protein phosphorylation plays a role in PIN3 trafficking to the plasma membrane.

Locus identifier	Primary Gene Symbol	Gene Model Description
AT1G73590	PIN-FORMED 1 (PIN1)	Encodes an auxin efflux carrier involved in shoot and root development. It is involved in the maintenance of embryonic auxin gradients. Loss of function severely affects organ initiation, pin1 mutants are characterised by an inflorescence meristem that does not initiate any flowers, resulting in the formation of a naked inflorescence stem. PIN1 is involved in the determination of leaf shape by actively promoting development of leaf margin serrations. In roots, the protein mainly resides at the basal end of the vascular cells, but weak signals can be detected in the epidermis and the cortex. Expression levels and polarity of this auxin efflux carrier change during primordium development suggesting that cycles of auxin build-up and depletion accompany, and may direct, different stages of primordium development. PIN1 action on plant development does not strictly require function of PGP1 and PGP19 proteins.
AT1G74660	MINI ZINC FINGER 1 (MIF1)	Encodes MINI ZINC FINGER 1 (MIF1) which has a zinc finger domain but lacks other protein motifs normally present in transcription factors. MIF1 physically interact with a group of zinc finger-homeodomain (ZHD) transcription factors, such as ZHD5 (AT1G75240), that regulate floral architecture and leaf development. Gel mobility shift assays revealed that MIF1 blocks the DNA binding activity of ZHD5 homodimers by competitively forming MIF1-ZHD5 heterodimers. Constitutive overexpression of MIF1 caused dramatic developmental defects, seedlings were non-responsive to gibberellin (GA) for cell elongation, hypersensitive to the GA synthesis inhibitor paclobutrazol (PAC) and abscisic acid (ABA), and hyposensitive to auxin, brassinosteroid and cytokinin, but normally responsive to ethylene.
AT1G77280		Protein kinase protein with adenine nucleotide alpha hydrolases-like domain
AT1G78100		F-box family protein;
AT2G03730	ACT DOMAIN REPEAT 5 (ACR5)	Member of a small family of ACT domain containing proteins. ACT domains are thought to be involved in amino acid binding.
AT2G14960	(GH3.1)	encodes a protein similar to IAA-amido synthases. Lines carrying an insertion in this gene are hypersensitive to auxin.
AT2G18690		unknown protein;
AT2G18980	Peroxidase 16	Peroxidase superfamily protein
AT2G23170	(GH3.3)	encodes an IAA-amido synthase that conjugates Asp and other amino acids to auxin in vitro.
AT2G25790		Leucine-rich receptor-like protein kinase family protein;
AT2G30040	MITOGEN-ACTIVATEDPROTEIN KINASE KINASE KINASE 14 (MAPKKK14)	member of MEKK subfamily
AT2G33310	AUXIN-INDUCED PROTEIN13 (IAA13)	Auxin induced gene, IAA13 (IAA13).
**AT2G34650**	**PINOID (PID)**	**Encodes a protein serine/threonine kinase that may act as a positive regulator of cellular auxin efflux, as a a binary switch for PIN polarity, and as a negative regulator of auxin signaling. Recessive mutants exhibit similar phenotypes as pin-formed mutants in flowers and inflorescence but distinct phenotypes in cotyledons and leaves. Expressed in the vascular tissue proximal to root and shoot meristems, shoot apex, and embryos. Expression is induced by auxin. Overexpression of the gene results in phenotypes in the root and shoot similar to those found in auxin-insensitive mutants. The protein physically interacts with TCH3 (TOUCH3) and PID-BINDING PROTEIN 1 (PBP1), a previously uncharacterized protein containing putative EF-hand calcium-binding motifs. Acts together with ENP (ENHANCER OF PINOID) to instruct precursor cells to elaborate cotyledons in the transition stage embryo. Interacts with PDK1. PID autophosphorylation is required for the ability of PID to phosphorylate an exogenous substrate. PID activation loop is required for PDK1-dependent PID phosphorylation and requires the PIF domain. Negative regulator of root hair growth. PID kinase activity is critical for the inhibition of root hair growth and for maintaining the proper subcellular localization of PID.**
AT2G35930	PLANT U-BOX 23 (PUB23)	Encodes a cytoplasmically localized U-box domain containing E3 ubiquitin ligase that is involved in the response to water stress and acts as a negative regulator of PAMP-triggered immunity.
AT2G35980	YELLOW-LEAF-SPECIFIC GENE 9 (YLS9)	Encodes a protein whose sequence is similar to tobacco hairpin-induced gene (HIN1) and Arabidopsis non-race specific disease resistance gene (NDR1). Expression of this gene is induced by cucumber mosaic virus, spermine and during senescence. The gene product is localized to the chloroplast.
AT2G36220		unknown protein
AT2G39370		unknown protein;
AT2G39700	EXPANSIN A4 (EXPA4)	putative expansin.
AT2G40540	POTASSIUMTRANSPORTER 2 (KT2)	putative potassium transporter AtKT2p (AtKT2) mRNA,
AT2G41100	TOUCH 3 (TCH3)	encodes a calmodulin-like protein, with six potential calcium binding domains. Calcium binding shown by Ca(2+)-specific shift in electrophoretic mobility. Expression induced by touch and darkness. Expression may also be developmentally controlled. Expression in growing regions of roots, vascular tissue, root/shoot junctions, trichomes, branch points of the shoot, and regions of siliques and flowers.
AT2G41380		S-adenosyl-L-methionine-dependent methyltransferases superfamily protein;
AT2G42430	LATERAL ORGANBOUNDARIES-DOMAIN 16 (LBD16)	LOB-domain protein gene LBD16. This gene contains one auxin-responsive element (AuxRE).
AT2G42440		Lateral organ boundaries (LOB) domain family protein; CONTAINS InterPro DOMAIN/s: Lateral organ boundaries, LOB (InterPro:IPR004883)
AT2G42870	PHY RAPIDLY REGULATED1 (PAR1)	Encodes PHYTOCHROME RAPIDLY REGULATED1 (PAR1), an atypical basic helix-loop-helix (bHLP) protein. Closely related to PAR2 (At3g58850). Up regulated after simulated shade perception. Acts in the nucleus to control plant development and as a negative regulator of shade avoidance response. Functions as transcriptional repressor of auxin-responsive genes SAUR15 (AT4G38850) and SAUR68 (AT1G29510).
AT2G43590		Chitinase family protein
AT2G45400	(BEN1)	involved in the regulation of brassinosteroid metabolic pathway
AT2G45420	LOB DOMAIN-CONTAINING PROTEIN 18 (LBD18)	LOB domain-containing protein 18 (LBD18)
AT2G47130		NAD(P)-binding Rossmann-fold superfamily protein
AT2G47140		NAD(P)-binding Rossmann-fold superfamily protein
**AT2G47260**	**WRKY DNA-BINDING PROTEIN 23 (WRKY23)**	**Encodes a member of WRKY Transcription Factor; Group I. Involved in nematode feeding site establishment.**
AT3G02885	GAST1 PROTEINHOMOLOG 5 (GASA5)	GAST1 protein homolog 5 (GASA5); INVOLVED IN: response to gibberellin stimulus;
AT3G07010	Probable pectate lyase 8	Pectin lyase-like superfamily protein
AT3G07390	AUXIN-INDUCED IN ROOTCULTURES 12 (AIR12)	isolated from differential screening of a cDNA library from auxin-treated root culture. sequence does not show homology to any known proteins and is predicted to be extracellular.
AT3G09280		unknown protein
AT3G13380	Serine/threonine-protein kinase BRI1-like 3	Similar to BRI, brassinosteroid receptor protein.
AT3G15540	INDOLE-3-ACETIC ACIDINDUCIBLE 19 (IAA19)	Primary auxin-responsive gene. Involved in the regulation stamen filaments development.
AT3G16420	AT3G16420; AT5G54490 PINOID-BINDING PROTEIN 1 (PBP1)	Encodes a PINOID (PID)-binding protein containing putative EF-hand calcium-binding motifs. The interaction is dependent on the presence of calcium. mRNA expression is up-regulated by auxin. Not a phosphorylation target of PID, likely acts upstream of PID to regulate the activity of this protein in response to changes in calcium levels.
AT3G18560		unknown protein;
AT3G19200		unknown protein;
AT3G20830		AGC (cAMP-dependent, cGMP-dependent and protein kinase C) kinase family protein;
AT3G21700	(SGP2)	Monomeric G protein. Expressed in root epidermal cells that are destined to become atrichoblasts. Also expressed during pollen development and in the pollen tube tip.
AT3G23030	INDOLE-3-ACETIC ACID INDUCIBLE 2 (IAA2)	auxin inducible gene expressed in the nucleus
AT3G26760		NAD(P)-binding Rossmann-fold superfamily protein
AT3G28850		Glutaredoxin family protein

Locus identifier	Primary Gene Symbol	Gene Model Description
AT3G42800		unknown protein
AT3G49700	1-AMINOCYCLOPROPANE-1-CARBOXYLATE SYNTHASE 9 (ACS9)	encodes a a member of the 1-aminocyclopropane-1-carboxylate (ACC) synthase (S-adenosyl-L-methionine methylthioadenosine- lyase, EC 4.4.1.14) gene family. Mutants produce elevated levels of ethylene as etiolated seedlings.
AT3G50660	DWARF 4 (DWF4)	Encodes a 22α hydroxylase whose reaction is a rate-limiting step in brassinosteroid biosynthetic pathway. The protein is a member of CYP90B gene family. CLM is an epi-allele with small, compressed rosette, reduced internode length, and reduced fertility, appears in selfed ddm mutant plants possibly due to loss of cytosine methylation. Transcripts accumulate in actively growing tissues, and GUS expression is negatively regulated by brassinosteroids. Localized in the endoplasmic reticulum. The in vitro expressed protein can perform the C-22 hydroxylation of a variety of C27-, C28- and C29-sterols. Cholesterol was the best substrate, followed by campesterol. Sitosterol was a poor substrate.
AT3G51410		Arabidopsis protein of unknown function (DUF241)
AT3G54000		Uncharacterised conserved protein UCP022260
AT3G54950	PATATIN-RELATED PHOSPHOLIPASE IIIBETA (pPLAIIIbeta)	Encodes pPLAIIIbeta, a member of the Group 3 patatin-related phospholipases. pPLAIIIbeta hydrolyzes phospholipids and galactolipids and additionally has acyl-CoA thioesterase activity. Alterations of pPLAIIIβ result in changes in lipid levels and composition.
AT3G55690		unknown protein;
AT3G55720		Protein of unknown function (DUF620)
AT3G58190	LATERAL ORGANBOUNDARIES-DOMAIN 29 (LBD29)	This gene contains two auxin-responsive element (AuxRE).
AT3G60550	CYCLIN P3;2 (CYCP3;2)	cyclin p3;2 (CYCP3;2);
AT3G60630	HAIRY MERISTEM 2(HAM2)	Belongs to one of the LOM (LOST MERISTEMS) genes: AT2G45160 (LOM1), AT3G60630 (LOM2) and AT4G00150 (LOM3).<br>LOM1 and LOM2 promote cell differentiation at the periphery of shoot meristems and help to maintain their polar organization.
AT3G60640	AUTOPHAGY 8G (ATG8G)	AUTOPHAGY 8G (ATG8G)
AT3G62100	INDOLE-3-ACETIC ACID INDUCIBLE 30 (IAA30)	Encodes a member of the Aux/IAA family of proteins implicated in auxin signaling. IAA30 lacks the conserved degron (domain II) found in many family members. IAA30 transcripts are induced by auxin treatment and accumulate preferentially in the quiescent center cells of the root meristem. Overexpression of IAA30 leads to defects in gravitropism, root development, root meristem maintenance, and cotyledon vascular development. Target of LEC2 and AGL15. Promotes somatyic embryogenesis.
AT3G63440	CYTOKININOXIDASE/DEHYDROGENAS E 6 (CKX6)	This gene used to be called AtCKX7. It encodes a protein whose sequence is similar to cytokinin oxidase/dehydrogenase, which catalyzes the degradation of cytokinins.
AT4G00080	UNFERTILIZED EMBRYOSAC 11 (UNE11)	unfertilized embryo sac 11 (UNE11)
AT4G01870		tolB protein-related
AT4G11280	1-AMINOCYCLOPROPANE-1-CARBOXYLIC ACID (ACC) SYNTHASE 6 (ACS6)	encodes a a member of the 1-aminocyclopropane-1-carboxylate (ACC) synthase (S-adenosyl-L-methionine methylthioadenosine- lyase, EC 4.4.1.14) gene family
AT4G12410		SAUR-like auxin-responsive protein family ; CONTAINS InterPro DOMAIN/s: Auxin responsive SAUR protein (InterPro:IPR003676); BEST Arabidopsis thaliana protein match is: SAUR-like auxin-responsive protein family (TAIR:AT4G22620.1); Has 1137 Blast hits to 1128 proteins in 26 species: Archae - 0; Bacteria - 0; Metazoa - 0; Fungi - 0; Plants - 1136; Viruses - 0; Other Eukaryotes - 1 (source: NCBI BLink).
AT4G12720	Nudix hydrolase 7 (NUDT7)	Encodes a protein with ADP-ribose hydrolase activity. Negatively regulates EDS1-conditioned plant defense and programmed cell death.
AT4G13180		NAD(P)-binding Rossmann-fold superfamily protein).
AT4G13195	CLAVATA3/ESR-RELATED44 (CLE44)	Belongs to a large gene family, called CLE for CLAVATA3/ESR-related, encoding small peptides with conserved carboxyl termini. The C-terminal 12 amino acid sequence of CLE44 is identical to that of a dodeca peptide (TDIF, tracheary element differentiation inhibitory factor) isolated from Arabidopsis and functions as a suppressor of plant stem cell differentiation. TDIF sequence is also identical to the C-terminal 12 amino acids of CLE41 (At3g24770).
AT4G14130	XYLOGLUCANENDOTRANSGLUCOSYLAS E/HYDROLASE 15 (XTH15)	xyloglucan endotransglycosylase-related protein (XTR7)
AT4G14560	INDOLE-3-ACETIC ACIDINDUCIBLE (IAA1)	auxin (indole-3-acetic acid) induced gene (IAA1) encoding a short-lived nuclear-localized transcriptional regulator protein.
AT4G17350		DOMAIN/s: Pleckstrin-like, plant (InterPro:IPR013666), Protein of unknown function DUF828 (InterPro:IPR008546), Pleckstrin homology (InterPro:IPR001849
AT4G22530		S-adenosyl-L-methionine-dependent methyltransferases superfamily protein
AT4G22620		SAUR-like auxin-responsive protein family
AT4G22780	ACT DOMAIN REPEAT 7(ACR7)	Member of a family of ACT domain containing proteins . ACT domains are involved in amino acid binding .
AT4G27260	(WES1)	encodes an IAA-amido synthase that conjugates Asp and other amino acids to auxin in vitro. Lines carrying insertions in this gene are hypersensitive to auxin.
AT4G27280		Calcium-binding EF-hand family protein;
AT4G28640	INDOLE-3-ACETIC ACIDINDUCIBLE 11 (IAA11)	Auxin induced gene, IAA11 (IAA11).
AT4G30420		nodulin MtN21 /EamA-like transporter family protein
AT4G31910		HXXXD-type acyl-transferase family protein
AT4G32280	INDOLE-3-ACETIC ACIDINDUCIBLE 29 (IAA29)	Auxin inducible protein.
AT4G37290		unknown protein
AT4G37295		unknown protein
AT4G37590	NAKED PINS IN YUCMUTANTS 5 (NPY5)	A member of the NPY gene family (NPY1/AT4G31820, NPY2/AT2G14820, NPY3/AT5G67440, NPY4/AT2G23050, NPY5/AT4G37590). Involved in auxin-mediated organogenesis.
AT5G01840	OVATE FAMILY PROTEIN 1 (OFP1)	Encodes a member of the plant specific ovate protein family. Members of this family have been shown to bind to KNOX and BELL-like TALE class homeodomain proteins. This interaction may mediate relocalization of the TALE homeodomain from the nucleus to the cytoplasm. Functions as a transcriptional repressor that suppresses cell elongation.
AT5G02760		Protein phosphatase 2C family protein
AT5G04980		DNAse I-like superfamily protein
AT5G05160	REDUCED IN LATERAL GROWTH1 (RUL1)	Encodes a receptor-like kinase that activates secondary growth, the production of secondary vascular tissues.
AT5G06080	LOB DOMAIN-CONTAINING PROTEIN 33 (LBD33)	LOB domain-containing protein 33 (LBD33)
AT5G12050		unknown protein;
AT5G16110		unknown protein
AT5G17340		Putative membrane lipoprotein
AT5G18560	(PUCHI)	Encodes PUCHI, a member of the ERF (ethylene response factor) subfamily B-1 of ERF/AP2 transcription factor family. The protein contains one AP2 domain. There are 15 members in this subfamily including ATERF-3, ATERF-4, ATERF-7, and leafy petiole. PUCHI is required for morphogenesis in the early lateral root primordium of Arabidopsis. Expressed in early floral meristem (stage 1 to 2). Required for early floral meristem growth and for bract suppression. Triple mutant with bop1 and bop2 displays a strong defect in the determination of floral meristem identity with reduced LFY expression and the lack of AP1 expression.
AT5G26930	GATA TRANSCRIPTIONFACTOR 23 (GATA23)	Encodes a member of the GATA factor family of zinc finger transcription factors. Controls lateral root founder cell specification.
AT5G40540		Protein kinase superfamily protein

Locus identifier	Primary Gene Symbol	Gene Model Description
AT5G43700	AUXIN INDUCIBLE 2-11(ATAUX2-11)	Auxin inducible protein similar to transcription factors.
AT5G47370	(HAT2)	homeobox-leucine zipper genes induced by auxin, but not by other phytohormones. Plays opposite roles in the shoot and root tissues in regulating auxin-mediated morphogenesis.
AT5G48150	PHYTOCHROME A SIGNALTRANSDUCTION 1 (PAT1)	Member of GRAS gene family. Semi-dominant mutant has a reduced response to far-red light and appears to act early in the phytochrome A signaling pathway.
AT5G49448	AT5G49448	
AT5G51670		Protein of unknown function (DUF668)
AT5G52900		unknown protein
AT5G54490	PINOID-BINDING PROTEIN 1 (PBP1)	Encodes a PINOID (PID)-binding protein containing putative EF-hand calcium-binding motifs. The interaction is dependent on the presence of calcium. mRNA expression is up-regulated by auxin. Not a phosphorylation target of PID, likely acts upstream of PID to regulate the activity of this protein in response to changes in calcium levels.
AT5G54500	FLAVODOXIN-LIKEQUINONE REDUCTASE 1 (FQR1)	Encodes a flavin mononucleotide-binding flavodoxin-like quinone reductase that is a primary auxin-response gene.
AT5G54510	DWARF IN LIGHT 1 (DFL1)	Encodes an IAA-amido synthase that conjugates Ala, Asp, Phe, and Trp to auxin. Lines overexpressing this gene accumulate IAA- ASP and are hypersensitive to several auxins. Identified as a dominant mutation that displays shorter hypocotyls in light grown plants when compared to wild type siblings. Protein is similar to auxin inducible gene from pea (GH3).
AT5G57100		Nucleotide/sugar transporter family protein
AT5G57520	ZINC FINGER PROTEIN 2(ZFP2)	Encodes a zinc finger protein containing only a single zinc finger.
AT5G62280		Protein of unknown function (DUF1442)
AT5G64250		Aldolase-type TIM barrel family protein
AT5G65390	ARABINOGALACTANPROTEIN 7 (AGP7)	arabinogalactan protein 7 (AGP7)
AT5G67430		Acyl-CoA N-acyltransferases (NAT) superfamily protein
