# Supplementary material for: WRKY23 is a component of the transcriptional network mediating auxin feedback on PIN polarity
Source: PLoS Genet. 2018 Jan 29;14(1):e1007177. doi: 10.1371/journal.pgen.1007177 (PMC5805370; doi:10.1371/journal.pgen.1007177)
Supplement: S3 Table — (PDF) [file pgen.1007177.s007.pdf]

S3 Table

Primer	Sequence	Reference
PID_FOR	5 -TGCCGACTCTTTACGCTGAG-3	This study
PID_REV	5 -CTTCGGCGGCATAAAATCTGG-3	This study
WRKY23_FOR	5 -AGTCTCGGTAATGGTTGCTTTGG-3	Grunewald et al., 2013
WRKY23_REV	5 -TGTTGCTGCTGTTGGTGATGG-3	Grunewald et al., 2013
SALK_003943_FOR	5 -CGGTGGGTTTATCAACAAATG-3	This study
SALK_003943_REV	5 -TGTTGCTGCTGTTGGTGATGG-3	This study
SALK_38289_FOR	5 -CGATCACATTTTTCTTTTCGC-3	This study
SALK_38289_REV	5 -CGGTGGGTTTATCAACAAATG-3	This study
TUB-2_FOR	5 -ACTCGTTGGGAGGAGGAACT-3	Grunewald et al., 2013
TUB-2_REV	5 -ACACCAGACATAGTAGCAGAAATCAAG-3	Grunewald et al., 2013
IAA14_FOR	5 -CCTTCTAAGCCTCCTGCTAAAGC-3	Shkolnik-Inbar and Bar-Zvi, 2010
IAA14_REV	5 -CCGCTCTTCTGATTAGCCATAAC-3	Tejos et al., 2014
PIP5K1_FOR	5 -GGAACATTGTGAATCGAGGACTG-3	Tejos et al., 2014
PIP5K1_REV	5 -CCGTCTCGTCTCTCTACTTCTT-3	Tejos et al., 2014
PIP5K2_FOR	5 -ATGATGCGTGAACCGCTTG-3	Tejos et al., 2014
PIP5K2_REV	5 -TTCCATGCTGCAGGTTGAGCA-3	Tejos et al., 2014
